# A recurrent p.Arg92Trp variant in steroidogenic factor-1 (NR5A1) can act as a molecular switch in human sex development

**DOI:** 10.1093/hmg/ddw186

**Published:** 2016-07-04

**Authors:** Anu Bashamboo, Patricia A. Donohoue, Eric Vilain, Sandra Rojo, Pierre Calvel, Sumudu N. Seneviratne, Federica Buonocore, Hayk Barseghyan, Nathan Bingham, Jill A. Rosenfeld, Surya Narayan Mulukutla, Mahim Jain, Lindsay Burrage, Shweta Dhar, Ashok Balasubramanyam, Brendan Lee, Members of UDN, Marie-Charlotte Dumargne, Caroline Eozenou, Jenifer P. Suntharalingham, KSH de Silva, Lin Lin, Joelle Bignon-Topalovic, Francis Poulat, Carlos F. Lagos, Ken McElreavey, John C. Achermann

**Affiliations:** 1 Human Developmental Genetics, Institut Pasteur, Paris, 75724 France; 2 Department of Pediatrics, Endocrinology & Diabetes, Medical college of Wisconsin, Milwaukee, WI, USA; 3 Departments of Human Genetics, Pediatrics and Urology, David Geffen School of Medicine at UCLA, CA, USA; 4 Department of Pediatrics, Faculty of Medicine, University of Colombo, Colombo 08, Sri Lanka; 5 Genetics & Genomic Medicine, UCL Institute of Child Health, University College London, London, UK; 6 Department of Human Genetics, David Geffen School of Medicine at UCLA, CA, USA; 7 Division of Pediatric Endocrinology and Diabetes, Department of Pediatrics, Vanderbilt University, Nashville, TN, USA; 8 Genetic and Development Department, Institute of Human Genetics, CNRS, Montpellier, France; 9 Department of Endocrinology, Pontificia Universidad Católica de Chile, and Universidad San Sebastián, Santiago, Chile; 10 Department of Molecular and Human Genetics, Baylor College of Medicine, TX; 11 Department of Medicine, Division of Diabetes, Endocrinology and Metabolism, Baylor College of Medicine, Houston TX, USA; 12 Undiagnosed Diseases Network (members listed in Supplemental Material)

## Abstract

Cell lineages of the early human gonad commit to one of the two mutually antagonistic organogenetic fates, the testis or the ovary. Some individuals with a 46,XX karyotype develop testes or ovotestes (testicular or ovotesticular disorder of sex development; TDSD/OTDSD), due to the presence of the testis-determining gene, *SRY*. Other rare complex syndromic forms of TDSD/OTDSD are associated with mutations in pro-ovarian genes that repress testis development (e.g. *WNT4*); however, the genetic cause of the more common non-syndromic forms is unknown. Steroidogenic factor-1 (known as NR5A1) is a key regulator of reproductive development and function. Loss-of-function changes in NR5A1 in 46,XY individuals are associated with a spectrum of phenotypes in humans ranging from a lack of testis formation to male infertility. Mutations in *NR5A1* in 46,XX women are associated with primary ovarian insufficiency, which includes a lack of ovary formation, primary and secondary amenorrhoea as well as early menopause. Here, we show that a specific recurrent heterozygous missense mutation (p.Arg92Trp) in the accessory DNA-binding region of NR5A1 is associated with variable degree of testis development in 46,XX children and adults from four unrelated families. Remarkably, in one family a sibling raised as a girl and carrying this *NR5A1* mutation was found to have a 46,XY karyotype with partial testicular dysgenesis. These unique findings highlight how a specific variant in a developmental transcription factor can switch organ fate from the ovary to testis in mammals and represents the first missense mutation causing isolated, non-syndromic 46,XX testicular/ovotesticular DSD in humans.

## Introduction

Disorders of (or differences in) sex development (DSD) are defined as congenital conditions in which the development of chromosomal, gonadal or anatomical sex is atypical (1,2). Classic forms of DSD affect approximately one in every 2000 to 4000 people and may present in many ways, such as a baby with atypical genitalia where it cannot immediately be said whether the newborn is a boy or girl, or a teenage girl with primary amenorrhoea. Related conditions, such as hypospadias, are more prevalent, affecting up to one in 300 boys.

DSDs encompass a wide range of different aetiologies (1–3). Sex chromosome mosaicism (45,X/46,XY) and 46,XY DSD (gonadal dysgenesis, disorders of androgen synthesis and action) are relatively common causes, whereas most individuals with 46,XX forms of DSD have congenital adrenal hyperplasia (CAH; MIM: 202010). Importantly, a subset of individuals with a 46,XX karyotype has ovotestes or testes rather than an adrenal condition. These forms of ovotesticular DSD (OTDSD) and testicular DSD (TDSD) were historically known as “true hermaphroditism” and “XX males”, respectively, and need different approaches to counselling and management compared to CAH (2,3).

Our molecular understanding of the causes of OTDSD/TDSD is incomplete. Some individuals with OTDSD/TDSD have upregulation of genes involved in testis determination (e.g. *SRY* MIM: 400045*, SOX9* MIM: 278850*, SOX3* MIM: 300833), whereas others have reduced function of genes expressed in the developing ovary that repress testis development (e.g., *RSPO1* MIM: 610644*, WNT4* MIM: 611812; 4–9). To date, coding mutations in single genes causing non-syndromic OTDSD/TDSD have not been described.

NR5A1, also known as steroidogenic factor-1 (SF-1) is a nuclear receptor transcription factor with key roles in reproductive development and function (10). Loss-of-function changes in NR5A1 are associated with a wide spectrum of conditions including gonadal (testicular) dysgenesis (MIM: 612965), hypospadias, and male factor infertility (MIM: 613957) in 46,XY individuals, as well as primary ovarian insufficiency (POI, MIM: 612964) in 46,XX women (11–15). Here, we show that a recurrent heterozygous point mutation in *NR5A1* is associated with OTDSD/TDSD development in 46,XX individuals in four families of different ancestry.

## Results

### Clinical phenotypes

**Figure 1.**
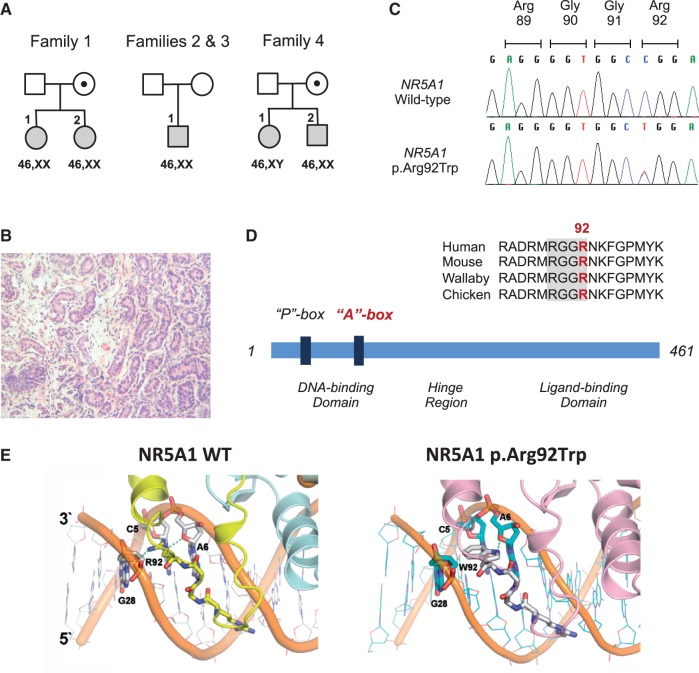
Identification and modelling of the NR5A1 p.Arg92Trp variant. (**A**) Shows the pedigrees of the four families with the affected members shaded. Squares denote individuals brought up as boys and circles denote individuals brought up as girls, with respective karyotypes shown below. (**B**) Shows testicular histology from individual 4.2 with 46,XX testicular DSD. (**C**) Shows sequence chromatograms of the wild-type and p.Arg92Trp *NR5A1*. (**D**) Shows the sequence alignment of the A-box motif in selected vertebrae, together with a schematic representation of the NR5A1 protein. (**E**) Shows the model of wild-type NR5A1 and p.Arg92Trp DNA-binding domain in complex with DNA. The RGGR motif of the A-box interacts with the minor groove of DNA. The basic arginine residue (Arg, R) in wild-type NR5A1 (left panel) is replaced by a neutral tryptophan residue (Trp, W) with a bulky indole side chain (right panel).

Family 1, of European-American ancestry, have two 46,XX children with ovotestes (Figure 1A; Table 1) . The first child (1.1) presented with atypical (“ambiguous”) genitalia at birth. Gonads were palpable in the right inguinal region and higher in the left labioscrotal fold. The karyotype was 46,XX (*SRY*-negative) and 17-hydroxyprogestone was low, excluding CAH. Gonadal biopsies showed dysgenetic testicular tissue. Vaginoscopy revealed a rudimentary vagina and uterine cervix. Following discussions with the parents, the female sex assignment was considered appropriate. Gonadal histology showed a left dysgenetic testis and right ovotestis, with a 46,XX karyotype in this tissue. A diagnosis of OTDSD was made. A second child (1.2) presented with similar genitalia at birth (Figure 1A; Table 1). Investigations revealed a 46,XX karyotype, intra-abdominal gonads and a uterus. Gonadal histology showed bilateral ovotestes, suggesting a diagnosis of familial OTDSD. The mother had no known androgenization but had menopause at the age of 40 years.

**Table 1.** Overview of the genetic and phenotypic features of children and adults with ovotesticular and testicular DSD

Individual	1.1	1.2	2.1	3.1	4.1	4.2
Karyotype	46,XX	46,XX	46,XX	46,XX	46,XY	46,XX
*SRY*	–	–	–	–	+	–
NR5A1	p.Arg92Trp	p.Arg92Trp	p.Arg92Trp	p.Arg92Trp	p.Arg92Trp	p.Arg92Trp
Inheritance	Maternal	Maternal	*de novo*	^a^*de novo*	Maternal	Maternal
Sex assignment	Female	Female	Male	Male	Female	Male
Presentation	“Ambiguous” genitalia	“Ambiguous” genitalia	Small penis; retractile testes	Small testes; low testosterone	Mild clitoromegaly; labial fusion	Penoscrotal hypospadias
Gonad position	L: inguinoscrotal; R: inguinal	Intra-abdominal	Scrotal (retractile)	Scrotal	Inguinal	Scrotal
Gonad histology	L: dysgenetic testis; R: ovotestis	^b^ Bilateral ovotestes	^c^ N/A	N/A	Dysgenetic testes	Dysgenetic testes
Uterus	R: hemiuterus	Uterus	No uterus	No uterus	No uterus	No uterus
DIagnosis	46,XX ovotesticular DSD	46,XX ovotesticular DSD	46,XX testicular DSD	46,XX testicular DSD	46,XY partial gonadal dysgenesis	46,XX testicular DSD

^a^inferred from paternal haplotype (see Supplemental data and Fig. S2);

^b^histology reported primitive ova structures;

^c^ultrasound reported two testes with normal colour Doppler blood flow/spectral waveforms and biochemistry was consistent with the presence of testicular tissue.

N/A. not available.

Family 2, of Latino/Hispanic ancestry, have a boy (2.1) who was referred at seven months of age because of a small penis that was partly buried in suprapubic fat (stretched length 2.8cm, typical range 4.3cm +/− 0.8) (Figure 1A; Table 1). Both gonads were palpable at the time. At 9 months of age, chromosome analysis and ultrasound were ordered as the gonads were no longer palpable. A 46,XX karyotype (*SRY*-negative) was observed and two structures in the inguinal regions (1.3 × 0.5 × 0.7cm; 0.9 × 0.5 × 0.7cm) that had morphological and Doppler appearances of testes. Anti-Müllerian Hormone (AMH, MIS) (478 pmol/l [342–594]) and inhibin B (85 pg/ml [40–630]) concentrations were within normal ranges for a child with testes and a good testosterone response was seen following three days stimulation with human chorio-gonadotropin (basal <0.1 nmol/l, peak 15.1 nmol/l). No testicular biopsy was done. The diagnosis of 46,XX TDSD was made.

Family 3, of African and African-American ancestry, have a son (3.1) who presented with small testes and low testosterone at 16 years of age (Figure 1A; Table 1). He was on testosterone supplementation inconsistently through his late teens and 20s. At 30 years of age, he was found to have bilateral atrophic testes (less than 5cc) and small phallus (5–6 cm stretched length). Genetic evaluation showed a 46,XX karyotype (*SRY*-negative) with a normal microarray. Endocrine evaluation showed primary testicular failure, based on very low testosterone (34 ng/dL [300–1080]), raised gonadotrophins (FSH (25 mIU/mL [1.3–11.4]) and LH (15 mIU/ml [1.2–7.8])), and low AMH (0.657 pmol/l [15–219.2]) and inhibin B (<10 pg/ml [40–450]). Adrenal function and sex-hormone binding globulin levels were normal. Testosterone levels remained low following a 4-day hCG stimulation test (testosterone 46 ng/dL, androstenedione 0.272 ng/ml [0.33–1.34], and dihydrotestosterone 55 pg/ml [112–955]). Semen analysis showed azoospermia. The diagnosis of 46,XX TDSD was made.

Family 4, of South Asian ancestry, have an older girl (4.1) who was seen in six years of age because of labial fusion and mild clitoral enlargement. The karyotype was 46,XY (*SRY*-positive) (Figure 1A; Table 1). Gonads were palpable in the inguinal region, which were dysgenetic testes on histology. No uterus was found and a diagnosis of partial gonadal (testicular) dysgenesis was made. A younger brother (4.2) had severe penoscrotal hypospadias but palpable gonads in the scrotum. Biopsy of the right testis showed dysgenetic testicular tissue (Figure 1B). Surprisingly, the karyotype was 46,XX (*SRY*-negative), suggesting a diagnosis of 46,XX TDSD (Figure S1). The mother has no reported androgenisation but has had very irregular periods all her life. This family therefore has a 46,XY girl with partial gonadal dysgenesis and a 46,XX boy with TDSD who are siblings.

### NR5A1 p.Arg92Trp mutation associated with 46,XY DSD and 46,XX OTDSD/TDSD

In families 1, 2 and 3, whole exome (1 & 2) and whole genome sequencing (3), respectively, revealed a heterozygous *NR5A1* mutation p.Arg92Trp (p.R92W, c.274C > T) as the best candidate for the phenotype (Figure 1A and C; Table 1). In Family 4 direct sequencing of *NR5A1* revealed the same heterozygous mutation in the 46,XX TDSD boy and in his older sister who had 46,XY partial gonadal dysgenesis. This p.Arg92Trp change was inherited from the mother in families 1 and 4, but was *de novo* in family 2. In family 3, the mutation was not present in the father. The child’s mother was deceased and a DNA sample was not available for sequencing. However, haplotype reconstruction by direct comparison of the genotypes in the affected child and father indicated a strong likelihood of the variant being *de novo* on the paternal allele based on direct phasing in the patient and evaluation of the rare paternal haplotype shared by both the proband and his father (Supplementay Material, Results and Fig. S2). The p.Arg92Trp variant was not identified in NR5A1 in more than 800 known fertile individuals and was absent from >70,000 individuals present in public databases. Moreover, our families were unrelated and came from geographically distinct regions, excluding a founder effect for a rare variant. All 46,XX children were *SRY*-negative and exome or genome sequencing in family 1, subject 2.1 and subject 3.1 did not show any other likely pathogenic causes. Furthermore in family 1, a hidden Y chromosome mosacism was excluded by both FISH analysis and the absence of *SRY* amplification by PCR using DNA from gonadal tissue.

### *In silico* analysis predicts a disruptive effect of p.Arg92Trp on DNA binding

The p.Arg92Trp mutation affects a highly conserved amino acid in the RGGR motif of the “A-box” of NR5A1 (Figure 1D). This motif interacts with the minor groove of DNA to stabilize the monomeric binding by NR5A1 (Figure 1E). The mutation replaces a polar arginine with a bulky tryptophan and likely alters hydrogen bonding between NR5A1 and DNA in this region (Figure1E) (see also Supplementary Material, Figures S3 and S4).

### NR5A1 is expressed in somatic cells of the early embryonic ovary

**Figure 2.**
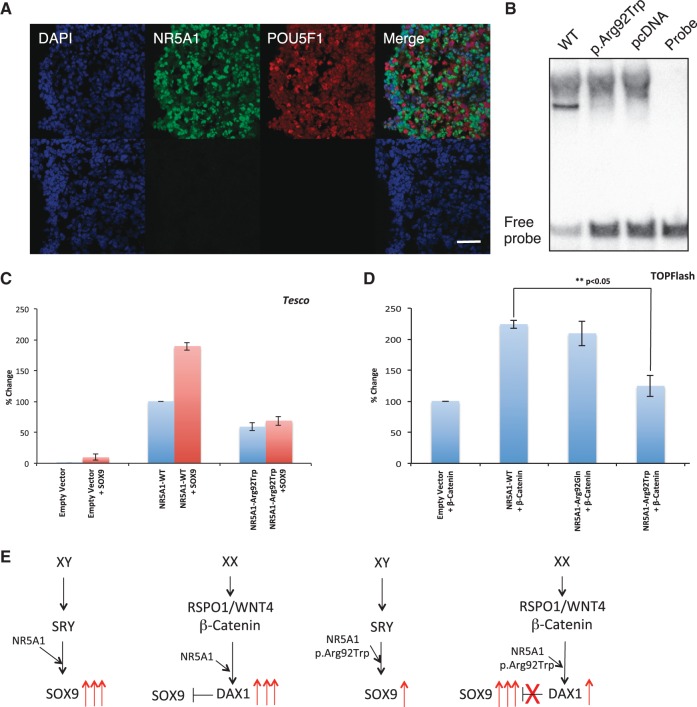
Expression, functional analyses and model of NR5A1 p.Arg92Trp acting as a cell fate switch. (**A**) Shows expression of NR5A1 in somatic cells of the developing ovary with germ cells shown by POU5F1 (OCT4) (9 wpc) (upper panel). Negative controls without primary antibody are shown below. Nuclei are stained blue with DAPI. Scale bar denotes 50 µm. (**B**) Demonstrates that the mutant NR5A1 p.Arg92Trp lacks the ability to bind to a known consensus target sequence. (**C**) Shows a reduced ability of the NR5A1 mutant protein to activate reporter activity using the *Tesco* enhancer element as a target in HEK 293-T cells. (**D**) HEK 293-T cells were transfected with multimerized TCF/LEF1 binding sites containing the luciferase reporter construct (TOPFlash). Co-transfection with the vectors pCS**β**-catenin with pCMX-NR5A1 WT, pCMX-NR5A1p.Arg92Gln or pCMX-NR5A1p.Arg92Trp showed that in contrast to both the NR5A1 WT and NR5A1 p.Arg92Gln proteins the NR5A1 p.Arg92Trp showed a reduced ability to synergise with **β**-catenin to up-regulate reporter gene activity. Similar results were obtained using KGN cells (data not shown). All results are expressed as mean +/- S.D. of at least three experiments. (**E**) shows a potential model based on the *in vitro* data. Left: in a 46,XY individual NR5A1 synergises with SRY to upregulate *SOX9* expression leading to testis formation. Left centre: in 46,XX individuals, NR5A1 syngerises with **β**-catenin to upregulate the expression of anti-testis genes (e.g. DAX-1/*NR0B1*). Right centre: In the 46,XY DSD case (4.1) the p.Arg92Trp mutant shows a reduced ability to up-regulate *SOX9* gene expression leading to a lack of testis formation. Right: In a 46,XX child with TDSD/OTDSD, the same mutant shows reduced ability to synergise with **β**-catenin to up-regulate the expression of anti-testis genes. As a consequence of this lack of repression, the expression of pro-testis genes (e.g. *SOX9*) leads to testis formation.

In order to assess whether *NR5A1* is expressed in the early human ovary, studies were performed using foetal tissue between 6–10 wpc. In contrast to the mouse, where *Nr5a1* is expressed specifically in the somatic cells of the early testis and only trace expression is observed in the early ovary (16,17), we found that the expression of human *NR5A1* was similar in the ovary and testis and significantly greater than in control tissue (data not shown). NR5A1 protein localized specifically to the nuclei of somatic cells in the ovary (Figure 2A). These data show that NR5A1 is expressed in ovarian somatic cells that can regulate cell fate at an early stage.

### P.Arg92Trp alters the biological activity of the protein

We hypothesized that the p.Arg92Trp mutation could be associated either with inappropriate activation of testis-specific pathways in the ovary, or with disruption of the pathways that oppose testis development and maintain ovarian integrity. In general, the p.Arg92Trp NR5A1 mutant showed loss of binding to a consensus NR5A1 response element and reduced activation of several minimal promoters (*Amh*, *Cyp11a1*) and enhancers (*Sox9 Tesco*) involved in testis development (Figures 2B and 2C, and Supplementary Material, Figure S5) consistent with 46,XY partial gonadal dysgenesis seen in the girl (4.1). Furthermore, the p.Arg92Trp NR5A1 mutant was unable to upregulate endogenous *Sox9* expression in mouse XX *Sf1-GFP*+ gonadal supporting cells compared to the WT NR5A1 protein (Supplementary Material, Figure S6). We recently described a homozygous p.Arg92Gln mutation in a girl with adrenal insufficiency and no ovotestis (18). We assayed the ability of these two different mutations to influence the pro-ovary canonical WNT/RSPO1 signalling pathway using the TOP-Flash reporter system. β- catenin has been shown to interact functionally with NR5A1 to activate target genes synergistically, including *Nr0b1* (Dax-1). In contrast to p.Arg92Gln, the p.Arg92Trp NR5A1 mutant showed selective loss of synergy with β-catenin (Figure 2D). Therefore, the phenotype in the 46,XX children with the p.Arg92Trp variant may be due to altered expression of ovarian factors that oppose testis development.

## Discussion

When a child presents with potential differences in sex development, making a specific diagnosis can have important implications for management and for supporting the family and young person appropriately (2,3,19). Most children with a 46,XX karyotype and atypical genitalia will have a form of CAH, usually due to 21-hydroxylase deficiency, and will require urgent investigation and adrenal steroid replacement. However, less common conditions such as ovotesticular DSD should also be considered in a baby with atypical genitalia, or testicular DSD in a boy with a 46,XX karyotype. Men with 46,XX testicular DSD typically present with small testes, testosterone insufficiency and infertility. Such individuals are often diagnosed through infertility clinics as several Y chromosomal genes are required to support spermatogenesis and two X chromosomes is deleterious for germ cell development in the XX testis. Experienced genetic counselling and support are needed to help the young person understand their condition and how it might affect their future. Given the increasingly recognized role of NR5A1 in many reproductive phenotypes a detailed family history is needed as other family members might be affected and could benefit from endocrine evaluation and appropriate counselling.

In humans, the embryonic gonad remains “bipotential” until around 6 wpc. In the developing testis, expression of the testis-determining gene *SRY* leads to upregulation of *SOX9* and subsequent stimulation of testis-specific pathways (Figure 2E; 20). Although poorly understood, it is emerging that many ovary-specific genes and pathways exist (21). Some of these signalling pathways, such as R-spondin-1 and WNT4, modulate their effects via β-catenin to “oppose” testis development by attenuating factors such as SOX9 (9,22,23).

NR5A1 clearly plays an important role in supporting testis development at multiple stages and loss of NR5A1 is associated with a wide range of testicular phenotypes. The relative phenotypes in Families 2 and 3 point to a differential ability of the NR5A1 mutant in sustaining in Sertoli and Leydig cell function early in human embryological development vs. later in postnatal life. Both subjects had evidence of testicular descent and absent Müllerian structures supporting functional Sertoli and Leydig cell activity. However, the subject in family 3 had no endocrinological evidence of such function in adult life (no inhibin B, AMH, and negative HCG stimulation) supporting the adult requirement for NR5A1 for maintaining Sertoli and Leydig cell function. The p.Arg92Trp mutant NR5A1 also likely disrupts pro-testis pathways to some extent given the results of functional studies as well as the partial testicular dysgenesis seen in the 46,XY girl in Family 4.

In contrast to the effects on testis development pathways, we suggest that the p.Arg92Trp NR5A1 variant switches organ fate from ovary to testis through disruption of ovary-specific pathways that would normally oppose testis development, rather than by activating testis pathways directly (Figure 2E). Indeed, our data show that, in contrast to the mouse model, NR5A1 is expressed in the somatic cells of the developing human ovary, and the p.Arg92Trp NR5A1 mutant shows a reduced ability to interact with β-catenin to synergistically activate reporter gene activity (16,17,24). Of note, a similar effect was not seen with the p.Arg92Gln mutant (Figure 2D), an NR5A1 variant which has been reported recently in a 46,XX girl without ovotestes (18). The expression of *Nr5a1* in the mouse embryonic gonad is sexually dimorphic. In contrast to its continuous expression in the mouse embryonic testis, *Nr5a1* transcripts are absent during the period of ovarian formation between E13.5-E16.5 (25). The absence of expression during ovarian formation suggests that a mouse model of the p.Arg92Trp mutant may not generate the same phenotype in XX mice and furthermore there is no guarantee that the p.Arg92Trp substitution will be pathogenic in the context of the mouse polypeptide.

Phenotype expression can be variable with NR5A1, as is often the case for developmental disorders. Children (46,XX) harbouring the p.Arg92Trp mutation had a phenotypic spectrum ranging from atypical (“ambiguous”) genitalia and a uterus to a phenotypic male with testes. Moreover, the mothers in families 1 and 4 transmitted the variant but had mild potential features. Possibly a second genetic variant could have accounted for variable penetrance, but no obvious factors were identified by exome sequencing. In addition, identification of this specific p.Arg92Trp change in multiple members of unrelated families of diverse ancestry suggests that it is the primary genetic driver of the condition. Sex development is exquisitely sensitive to dose-dependent effects. If a critical threshold of testis-determining factor function (e.g., SOX9) is reached then the testis development pathway will result, and a testis (or ovotestis) will form. We suspect that critical thresholds have been reached in these children, whereas these have not in the mothers. Indeed, familial cases would *by definition* only be identified if the mother had mild features and maintained fertility (26–28).

This report highlights the complexity of human sex development, reveals subtle but potentially important differences with the mouse system and it reveals a novel genetic cause of non-syndromic testis development in 46,XX individuals. Remarkably, a single amino acid change in a key transcription factor can function as a bidirectional switch the choice of developmental fate of tissues in early embryonic life.

## Materials and Methods

Complete methods are described in detail in Supplementary Material, Materials and Methods.

Ethics committee approval and written informed consent was obtained (Supplementary Material, Methods). Exome sequencing and direct sequencing of *NR5A1* was performed as described in the Supplemental Methods. Control data for *NR5A1* was obtained from direct sequencing of healthy fertile controls and public variant databases (Supplementary Material, Methods). Models of wild-type, p.Arg92Gln NR5A1 and p.Arg92Trp NR5A1 (hSF-1, Uniprot Q13285) in complex with DNA were generated with MODELLER software (29) and the crystal structure of mouse Nr5a1 bound to the inhibin α-subunit promoter (PDBid 2FF0) as a template (Supplementary Material, Methods). Human embryonic and fetal tissues were obtained from the Human Developmental Biology Resource (www.hdbr.org) following ethical approval and with informed consent (see Supplementary Material, Methods). Binding of wild-type and mutant NR5A1 to a consensus NR5A1 DNA binding site (CCAAGGTCA) was assessed using electromobility shift assays. Transient gene activation studies of NR5A1 were performed for wild-type and mutant cDNA using a range of different putative NR5A1 targets (*Amh*, *Cyp11a1*, *Tesco*) in different cell lines (HEK, KGN, NT2D1, mouse embryonic stem cells; 23). Synergistic activation of the TOP-Flash reporter system by NR5A1 and β-catenin binding was studied as reported previously (24 and see Supplementary Methods).

## Supplementary Material

Supplementary Material is available at *HMG* Online.

Supplementary Data

Supplementary Data
