# A recurrent p.Arg92Trp variant in steroidogenic factor-1 (NR5A1) can act as a molecular switch in human sex development

**DOI:** 10.1093/hmg/ddw390

**Published:** 2016-12-27

**Authors:** Anu Bashamboo, Patricia A. Donohoue, Eric Vilain, Sandra Rojo, Pierre Calvel, Sumudu N. Seneviratne, Federica Buonocore, Hayk Barseghyan, Nathan Bingham, Jill A. Rosenfeld, Surya Narayan Mulukutla, Mahim Jain, Lindsay Burrage, Shweta Dhar, Ashok Balasubramanyam, Brendan Lee, Members of UDN, Marie-Charlotte Dumargne, Caroline Eozenou, Jenifer P. Suntharalingham, KSH de Silva, Lin Lin, Joelle Bignon-Topalovic, Francis Poulat, Carlos F. Lagos, Ken McElreavey, John C. Achermann

**Affiliations:** 1 Human Developmental Genetics, Institut Pasteur, Paris, 75724 France; 2 Department of Pediatrics, Endocrinology & Diabetes, Medical college of Wisconsin, Milwaukee, WI, USA; 3 Departments of Human Genetics, Pediatrics and Urology, David Geffen School of Medicine at UCLA, CA, USA; 4 Department of Pediatrics, Faculty of Medicine, University of Colombo, Colombo 08, Sri Lanka; 5 Genetics & Genomic Medicine, UCL Institute of Child Health, University College London, London, UK; 6 Department of Human Genetics, David Geffen School of Medicine at UCLA, CA, USA; 7 Division of Pediatric Endocrinology and Diabetes, Department of Pediatrics, Vanderbilt University, Nashville, TN, USA; 8 Genetic and Development Department, Institute of Human Genetics, CNRS, Montpellier, France; 9 Department of Endocrinology, Pontificia Universidad Católica de Chile, and Universidad San Sebastián, Santiago, Chile; 10 Department of Molecular and Human Genetics, Baylor College of Medicine, TX; 11 Department of Medicine, Division of Diabetes, Endocrinology and Metabolism, Baylor College of Medicine, Houston TX, USA; 12 Undiagnosed Diseases Network (members listed in Supplemental Material)

The author Marie-Charlotte Dumargne should have been included in the author list of the above article. This has been corrected above and in the original article (doi:10.1093/hmg/ddw186).

