# Supplementary material for: A recurrent p.Arg92Trp variant in steroidogenic factor-1 (NR5A1) can act as a molecular switch in human sex development
Source: Hum Mol Genet. 2016 Jul 4;25(16):3446–53. doi: 10.1093/hmg/ddw186 (PMC5179941; doi:10.1093/hmg/ddw186)
Supplement: Supplementary Data [file supp_25_16_3446__index.html]

A recurrent p.Arg92Trp variant in steroidogenic factor-1 (NR5A1) can act as a molecular switch in human sex development — A recurrent p.Arg92Trp variant in steroidogenic factor-1 (NR5A1) can act as a molecular switch in human sex development — Supplementary Data 

# A recurrent p.Arg92Trp variant in steroidogenic factor-1 (NR5A1) can act as a molecular switch in human sex development

## Supplementary Data

files

- Supplementary Data - docx file
- Supplementary Data - pdf file
