# Supplementary material for: A recurrent p.Arg92Trp variant in steroidogenic factor-1 (NR5A1) can act as a molecular switch in human sex development
Source: Hum Mol Genet. 2016 Jul 4;25(16):3446–53. doi: 10.1093/hmg/ddw186 (PMC5179941; doi:10.1093/hmg/ddw186)
Supplement: Supplementary Data [file supp_ddw186_Revision_Supplemental_NR5A1_HMG.docx]

**SUPPLEMENTAL MATERIALS AND METHODS**

**Ethical Approval and informed consent**

Ethical committee approval for exome and genome studies was obtained from the Comité de Protection des Personnes, Ile-de-France (N°IRB00003835) and the National Human Genome Research Institute Institutional Review Board (15-HG-0130). Local institutional review boards also agreed to the study and written informed consent was obtained from family members.

**Validation of karyotype, *SRY* gene and sequencing of *NR5A1***

Standard G-banded karyotype analysis was performed on cultured metaphase lymphocytes in relevant clinical genetics units using standard protocols. The presence or absence of *SRY* was assessed using fluorescence in-situ hybridization or by PCR of the *SRY* gene from patient DNA and visualization of the PCR products on an agarose gel. PCR primers used for *SRY* amplification were SRY F 5`-CAGTCCAGCTGTGCAAGAGA-3` and SRY R 5`-GCCATTTTTCGGCTTCAGTA-3`. The PCR conditions were 95C 5 min, followed by 37 cycles of 95C 30 sec, 60C 30 sec, 72C 30 sec. The 272 bp fragment of the *SRY* gene was visualized on a 2% agarose gel with ethidium bromide staining. Sanger sequencing of the *NR5A1* gene was performed using amplified DNA extracted via conventional techniques from peripheral-blood lymphocytes of each individual and sequenced in accordance with protocols described elsewhere.^1^

**Control data**

Databases screened for the potential occurrence of the *NR5A1* mutation in the population were dbSNP138 (http://www.ncbi.nlm.nih.gov/snp/), the ExAC database (http://exac.broadinstitute.org/), the Exome Variant Server (EVS; http://evs.gs.washington.edu/EVS/), and the 1000 Genomes Project database (<http://browser.1000genomes.org/index.html>), as well as our internal database (400 exome sequenced individuals, >1,000 fertile controls Sanger sequenced for *NR5A1*).

**Whole Exome and Genome sequencing**

Exome sequencing for family 1 was performed as described elsewhere.^2^ Briefly, exon enrichment was performed using Agilent SureSelect Human All Exon V4. Paired-end sequencing was performed on the Illumina HiSeq2000 platform with an average sequencing coverage of x50. Read files were generated from the sequencing platform via the manufacturer’s proprietary software. Reads were mapped using the Burrows–Wheeler Aligner and local realignment of the mapped reads around potential insertion/deletion (indel) sites was carried out with the GATK version 1.6. SNP and indel variants were called using the GATK Unified Genotyper for each sample. SNP novelty was determined against dbSNP138. Datasets were filtered for novel or rare (MAF<0.01) variants.

For family 3 whole genome sequencing (30X coverage) was performed on the proband and proband’s father were completed as part of the Undiagnosed Diseases Network (UDN) project at HudsonAlpha Institute for Biotechnology (Huntsville, AL, USA). Structural and single-nucleotide variant detection from BAM files was performed at Baylor College of Medicine. We performed structural variant detection utilizing BreakDancer v1.4.5^3^ and SVDetect v0.8^4^. We selected potential breakpoints that were supported by >5 and <90 reads in both methods and that were concordant using both methods. We filtered variation described in the Database of Genomic Variants^5^ and segmental duplications^6^. Potential candidate structural variants were reviewed in the Savant Genome Browser^7^ and in collaboration with Baylor-Miraca Genetics Laboratories where a prior clinical array had been completed ([www.bmgl.com](http://www.bmgl.com)). We performed variant calling using PLATYPUS v0.7.9.1^8^, and variant annotation was completed using CASSANDRA v13.10.01^9^. The genomes were also uploaded to CODIFIED Genomics (<http://codifiedgenomics.com/>) for further annotation. We evaluated coding and splice-site variants that were present at allele frequency <0.001 in the ExAC database v0.3 (<http://exac.broadinstitute.org/>) and the 1000 genomes project^10^. We evaluated for potential recessively inherited variants by evaluating for at least 2 coding variants in a given gene, one of which was inherited from the proband’s father, and for potential *de novo* variants by filtering for novel variants not present in the father.

**Modeling of human NR5A1 and mutant protein**

The model of human Steroidogenic Factor 1 (NR5A1) (hSF-1, Uniprot Q13285) and mutant p.Arg92Gln and p.Arg92Trp in complex with DNA were generated using the MODELLER program as implemented in the Build Homology Model module of Discovery Studio v2.1 software (Accelrys Inc., San Diego, USA).^11^ The crystal structures of ligand binding domain (LBD) of human SF-1/NR5A1 in complex with PIP3 (PDBid 4QRJ)^12^, DAX-1 (PDBid 3F5C),^13^ cofactor peptides such as TIF2 or SHP (PDBid 1YP0),^14^ and the DNA binding domain (DBD) of murine Sf-1 in complex with target inhibin alpha-subunit promoter fragment (PDBid 2FF0) were used as template.^15^ Zinc atoms were incorporated during modeling and residues 123-214 of hSF-1/NR5A1 were not included in the modeling process due to lack of an appropriate template. For each protein-DNA complex, a set of 100 models were constructed and the best model according to Modeller internal PDF score was subjected to a molecular minimization protocol using the CHARMM 22 force-field.^16^ The protocol consisted of 10,000 steps of steepest descent method, followed by 25,000 steps of conjugate gradient method to reach a final root-mean square (RMS) gradient of 0.001 kcal/mol/Å^2^. The overall quality of the final models was assessed by Ramachandran plot analysis using the RAMPAGE server and quality model assessments were performed using the ProSA-web server.^17, 18^ APBS software was used to calculate the spatial distribution of electrostatic potential on protein atoms using a two-dielectric implicit solvent model and the finite difference method to solve the Poisson-Boltzmann Equation.^19^ Hydrophobicity was calculated according to Eisenberg scale of hydrophobicity and mapped into the solvent accessible surface of each protein.^20^

**Construction of wild-type and mutant vectors**

NR5A1 expression vectors containing the p.Arg92Trp or p.Arg92Gln mutations were generated by site-directed mutagenesis (QuikChange, Stratagene) with the use of wild-type (WT) human *NR5A1* cDNA in a pCMX expression vector as a template.^1^ The entire coding sequence of all mutant plasmids was confirmed by direct sequencing prior to functional studies.

**Gel shift assay with biotinylated probe and in vitro translated (SF1) protein**

Wild-type and mutant NR5A1 were in vitro translated using the TNT system (Promega)**.** Control reaction was performed using the empty vector. Effective and equal production of wild type and mutated NR5A1 protein was validated by in-vitro translation in presence of S^35^ methionine followed by SDS-PAGE and quantified using phosphorimager (GE Healthcare life science). Protein binding to target DNA probes was assessed by the electrophoretic mobility shift assay (EMSA). The oligos used for the EMSA were Biot_SF1_BS_for (5' TEG-biotin): CCCCCCAAGGTCACCTTTGG and Biot_SF1_BS_rev (5'-TEG biotin) : CCAAAGGTGACCTTGGGGGG. In these experiments, 5' TEG-biotin oligos are hybridized (50 µg per oligos) in 10mM Tris ph7.5, 1mM EDTA, 50 mM NaCl. The tube of oligonucleotides was incubated in boiling water for 5 minutes and allowed to slowly cool to room temperature. The hybridized probe was purified on a 6% nondenaturing acrylamide gel and eluted overnight in 0.5 M ammonium acetate–10mM Magnesium acetate-5 mM EDTA–0.1% SDS buffer at 50°C and concentrated by ethanol precipitation. Probe was quantified using Qubit fluoroimager (InVitrogen). For the binding reaction, 2 μl of programed TNT lysate was mixed with 50 fmol of purrified probe and 2 μg of poly(dI-dC) in 20-μl final volume of binding buffer (20 mM HEPES [pH 7.9], 5% glycerol, 0.1 M KCl, 0.1mM ZnSO4, 0.5 mM DTT). The DNA-protein complexes were resolved by electrophoresis on a 6% polyacrylamide gel (19:1) in 0.5X Tris-borate-EDTA buffer at 4°C and visualized using the chemiluminescent nucleic acid detection module (PIERCE-thermoscientific ref: 89880) with no modification of the protocol.

**Transient gene expression assays**

Transient gene expression assays for the assessment of NR5A1 function were performed in 96-well plates (TPP) with the use of either human embryonic kidney (HEK) 293T cells, KGN, NT2D1 or a mouse embryonic stem cell line (E14), Fugene 6 transfection reagent (Roche no. 1 814 443), and a Dual-Luciferase reporter assay system (Promega) with pRLSV40 Renilla luciferase (Promega) expression as a marker of transfection efficiency. pCMX-WT or mutant NR5A1 expression vectors (10 ng/well) were co-transfected into HEK293T cells with reporters containing NR5A1 (SF-1) responsive minimal promoters (murine *Cyp11a1*, human *AMH*) or enhancers (murine *Tesco*) (10 ng/well). To assay the ability of NR5A1 proteins to modulate the canonical WNT pathway, we used the TOPFlash-TCF reporter plasmid, as previously described.^21, 22^ Cells were lysed 48 hr later, and luciferase assays were performed with the use of a FLUOstar Optima fluorescence microplate reader (BMG Labtech). All data were standardized for Renilla activity. Results are shown as the mean ± SEM of three independent experiments, each performed in triplicate.

**Immunohistochemistry**

Human fetal ovary tissue (9 weeks post conception) was provided with approval from the Human Developmental Biology Resource (HDBR, [www.hdbr.org](http://www.hdbr.org)). Following cryosection, tissue sections (12 µm) were fixed briefly in 4% PFA in TBS, rinsed in TBS and blocked in 1% BSA in TBS-Tween (0.5% Tween) for 1 hour before incubating overnight with mouse monoclonal anti-SF-1 (NR5A1) antibody (ThermoFisher #434200, 1:200 dilution) or rabbit polyclonal anti-POU5F1 (OCT4) antibody (Abcam #Ab19857, 1:200 dilution). Sections were washed in TBS-Tween and incubated for 1 hour with the relevant secondary antibodies: Alexa488 goat anti-mouse (Invitrogen, A11001; 1:400) and Alexa555 goat anti-rabbit (Invitrogen, A21429; 1:400), respectively. Nuclei were counterstained with DAPI (Sigma). Slides were washed and mounted using ProLong Gold Antifade Mountant (Lifetech, #P36930). Images were collected on a Zeiss LSM 710 confocal microscope (Carl Zeiss) and analyzed using Zeiss Zen 2009 and Image J.

**Micromass culture of female gonad total cells from SF1/eGFP transgenic mouse**

SF1/eGFP+ male mouse^23^ were mated overnight with wild type Swiss female, vaginal plugs (VP) were checked at noon and female with VP were kept in separate cages until E12.5 dpc. At E12.5 dpc pregnant females were killed by CO_2_ asphyxiation followed by cervical dislocation and embryos were removed from uterine horns and kept in Leibowitz’s L-15 medium (Gibco, Paisley, UK) before dissection. Age of SF1/eGFP+ embryos was calculated by counting tail somites (E12.5 dpc, 29 ±3 ts). Embryonic gonads were dissected under a binocular microscope and distinction between E12.5 dpc male and female gonads was done by presence of seminiferous cords in the male gonads. Mesonephroi were carefully removed from genital ridges using U100 Insulin needle. After dissection, isolated female genital ridges were incubated for 10 min in 0.05 % trypsin-EDTA (Gibco, Paisley, UK), reaction was stopped by adding 10% FCS and dissociation was completed by repeated pipetting. Trypsin-digested cells were pooled according to sex, filtered on a 35 uM nylon mesh and pelleted at 200xg for 10 min. Cells were resuspended carefully in a DMEM/F12 (1:1) + Glutamax culture medium (Gibco, Paisley, UK) containing 2 % FCS, ITS (Sigma-Aldrich, St. Louis, MO, USA), EGF 20 ng/mL and Pen./Strep. cocktail. Cells were seeded in 24 wells-plates at 100,000 to 200,000 total cells per well, and grown for 24 hours at 37°C, 5% CO_2_ before further manipulations.

**Transfection of SF1/eGFP cells culture and cell collection**

After 24h-culture, medium was replaced and transfection of pCDNA3 mCherry LIC cloning vector (6B) (Addgene: #30125) containing either WT *hNR5A1*, p.R92W *hNR5A1* or no insert (empty) was performed with JetPRIME reagent according to manufacturer’s instructions (Polyplus transfection, Illkirch, France). After 24h of culture, cells were incubated for 2 min in 0.05 % trypsin-EDTA and directly filtered in a FACS tube with 35µm cap filter and kept on ice until cell sorting. For each samples (WT *hNR5A1*, R92W *hNR5A1* or empty vector), SF1/eGFP+;mCherry- and SF1/eGFP+;mCherry+ were sorted using a FACS Aria III (BD Biosciences, San Jose, CA, USA) according to a previous protocol ^24^.

**RNA extraction and qPCR experiments**

Cells were directly collected by FACS in the lysis buffer provided in RNeasy plus Microkit (Qiagen, Courtaboeuf, France) and processed according to the protocol defined by the manufacturer. The quality and quantity of RNA was verified on a 2100 Bioanalyzer (Agilent, Santa Clara, CA, USA) and only sample with a RNA integrity number (RIN) above 6 were processed with the Quantitect reverse transcription kit (Qiagen). qPCR experiments were performed on a 7900ht fast real-time PCR system (Life Technologies) with cDNA samples prepared from 100 ng of total RNA and using the Taqman gene assays (Life Technologies) designed for *Nr5a1* (Mm00446826_m1), *Sox9* (Mm00448840_m1) and *Rpl19* (Reference gene - Mm02601633_g1). The expression of each gene was assayed in triplicate and retrieved as 2^(-ΔCt)^ relative to *Rpl19* reference gene, where ΔCt is mean Ct_(gene of interest)_ - mean Ct _(Rpl19)_. In order to strictly evaluate the effect of wild type and mutant NR5A1 transfection over endogenous gene expression in SF1/eGFP+ cells, we retrieved gene expression as a ratio between expression values in transfected cells (i.e. SF1/eGFP+;mCherry+) versus non-transfected cells (i.e. SF1/eGFP+;mCherry-).

**Haplotype reconstruction for family 3**

For haplotype reconstruction we included single nucleotide variants that had >20 read depth. We performed a direct comparison of genotypes in a 500kb interval surrounding the candidate variant and identified the shared haplotype (PAT1). The haplotype presumed to be shared between proband and his mother (MAT1) was reconstructed by removing the PAT1 haplotype from the proband’s haplotype. PAT2, the second paternal haplotype, was defined by removing PAT1 haplotype from the paternal genotype. Haplotypes were compared to founders in the African populations of the 1000 genomes (GWD n=131, LWK n=99, ASW n=61, ACB n=97, MSL n=85, ESN n=102)^25^. Phasing was completed by review of called reads in the Integrative Genomics Viewer (IGV).^26^

**SUPPLEMENTAL RESULTS**

**Haplotype Reconstruction for family 3**

Given that the proband’s mother was deceased and a sample was not available for sequencing, we attempted to determine whether the variant was inherited from his mother or *de novo* on the paternal allele by reviewing haplotypes in the interval. In the proband we observed phasing of the *NR5A1* variant with an intronic insertion (Chr9:127263295C>CT) present in both the proband and the proband’s father (Figure S2). The intronic insertion is present in the ExAC database with the highest frequency (0.027) in African populations. A variant at this site is not listed in the 1000 genomes phase 3 variant call file^25^, thus we proceeded to identify the paternal and maternal haplotypes for comparison to the 1000 genomes data to clarify phasing of the intronic insertion. We performed haplotype reconstruction by direct comparison of the genotypes in the proband and father and identified the shared haplotype (PAT1), the haplotype presumed to be shared between proband and his mother (MAT1) and PAT2, the second paternal haplotype in the interval surrounding the *NR5A1* variant (Supplemental Table 1). We next compared these haplotypes to phased data from founders in the African populations of the 1000 genomes (GWD n=131, LWK n=99, ASW n=61, ACB n=97, MSL n=85, ESN n=102)^25^ and identified individuals that shared the PAT1 and MAT1 haplotypes (Supplemental Table 2). The PAT1 haplotype is overall rare (GWD AF=0.004, LWK AF=0.005, ASW AF=0, ACB AF=0.01, MSL AF=0.012), but of note, more common in the Esan and Yoruban Nigerian population (ESN AF=0.0245), consistent with the reported paternal ethnicity. The MAT1 haplotype is also rare (GWD AF=0.019, LWK AF=0.020, ACB AF=0.021, MSL AF=0.018, ESN AF=0.005) but most common in the African American from the SW US population (ASW AF=0.025), which is consistent with the maternal ancestry. The PAT2 haplotype is more common across the studied populations (GWD AF=0.080, LWK AF=0.157, ASW AF=0.057, ACB AF=0.124, MSL AF=0.200, ESN AF=0.162). We observed reads demonstrating the intronic variants in all individuals with the PAT1 haplotype and none of the individuals with the MAT1 haplotype (Supplemental Table 2). In addition we observed read-pairs in 3 individuals that are heterozygous for PAT1 that phase the intronic insertion to the rare haplotype (Figure S2B) and there are no read-pairs that are inconsistent with this phasing in the evaluated individuals. We conclude that there is a strong likelihood of the variant being *de novo* on the paternal allele based on direct phasing in the proband and evaluation of the rare PAT1 haplotype shared by both the proband and his father in the 1000 genomes project data.

**Supplemental Figures**

**Figure S1.**

**Figure S2.**


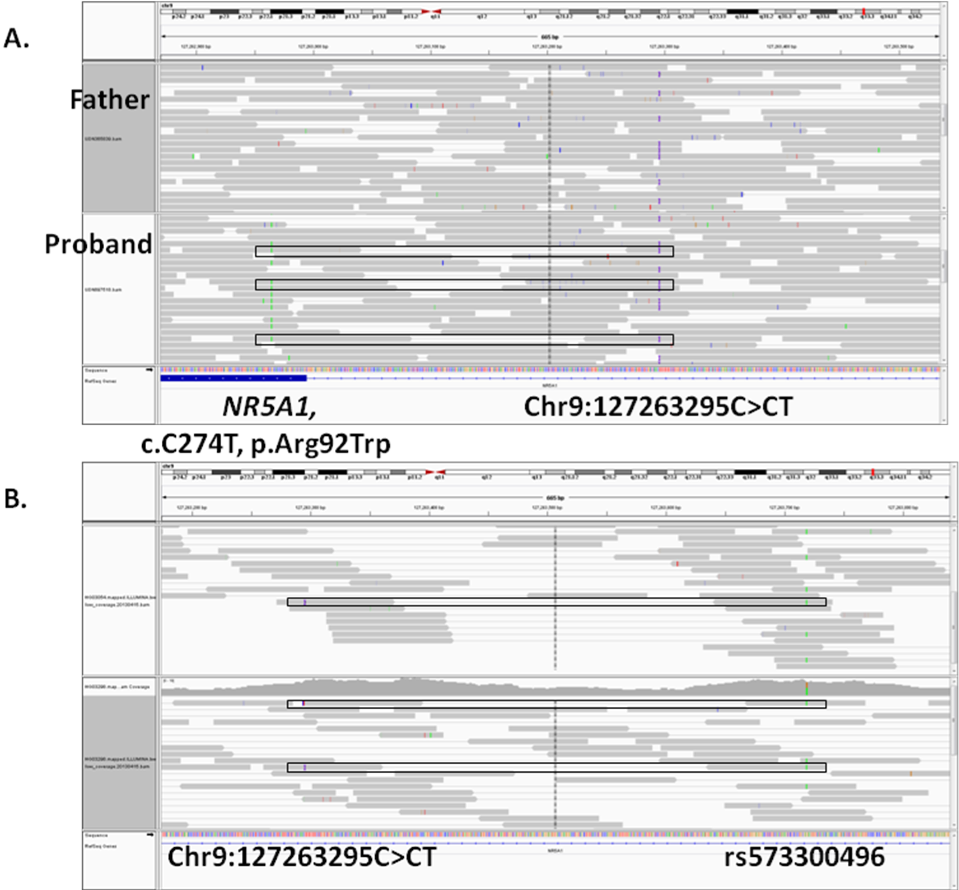


**Figure S3.**

**Figure S4.**

**Figure S5.**

**Figure S6.**

**SUPPLEMENTAL FIGURE LEGENDS**

**Figure S1. Karyotypes from family 4**, showing (A) 46,XY karyotype of the girl with partial gonadal dysgenesis (4.1) and (B) the 46,XX karyotype of the boy with TDSD (4.2).

**Figure S2. Read-Pair Evaluation Demonstrates Phasing of *NR5A1* Variant to Rare Paternal Haplotype in family 3.** Read-pairs are visualized in Integrative Genomics Viewer (IGV), and the reported coordinates are in hg19. 1A. *NR5A1* variant is present in the proband, but not his father. Both the proband and proband’s father have an intronic insertion (Chr9:127263295C>CT), and visual inspection identifies four read-pairs that phase the *NR5A1* variant to the intronic insertion. Haplotype reconstruction identified individuals from African populations of the 1000 genomes project that share this paternal haplotype. 1B. In addition to observing Chr9:127263295C>CT in all individuals with the paternal haplotype, read-pair analysis demonstrates direct phasing to rs573300496, a rare SNP that is present on the reconstructed paternal haplotype.

**Figure S3.** A) Schematic representation of human NR5A1-DNA complex model. The secondary structure of NR5A1 is shown in white cartoon with transparent surface. Depicted in different colors, reported binding partners are shown, such as PIP3 (PDBid 4QRJ),^12^ DAX-1 (PDBid 3F5C),^13^ and other cofactor peptides such as TIF2 or SHP (PDBid 1YP0).^18^ B) Snapshot of the human NR5A1 model DBD in complex with DNA , showing the different binding motifs of the core DBD which contains the zinc fingers and the A-box highlighting the RGGR motif in the C-terminal extension (CTE) that contains the R92 residue.

**Figure S4.** Comparison of electrostatics and hydrophobicity properties of wild-type and mutant human NR5A1. A) Electrostatic potential of wild-type and mutant NR5A1 plotted on the solvent-accessible surface at ±1kT/e. B) Eisenberg hydrophobicity scale mapped on the solvent-accessible surface of NR5A1. The red color indicates hydrophobic areas.

**Figure S5.** Transcriptional regulation of the *AMH* and *Cyp11a1* promoters by WT NR5A1 and NR5A1 p.Arg92Trp. Experiments were conducted using either human *AMH* promoter or mouse *Cyp11a1* promoter fused to luciferase as a reporter following transfection in HEK293-T cells. The data shown here represent the mean ± SEM of at least three independent experiments, each of which was performed in quadriplicate.

**Figure S6. *Sox9* NR5A1-dependent activation in mouse female SF1-GFP+ supporting cells is reduced with mutant p.R92W-NR5A1.** A) *Left* : Image of 48h-culture of female SF1GFP cells transfected with empty *pCDNA3 mCherry*, *WT-NR5A1::mCherry* or *R92W-NR5A1::mCherry*. Double fluorescent cells (i.e. GFP+/mCherry+) are indicated with a white arrow. *Right* : flow cytometry graphs representing the composition of GFP/mCherry cells. GFP+/mCherry- and GFP+/mCherry- were sorted in gate P3 and P4, respectively. B) qPCR comparison of mouse *Sox9* gene expression in female SF1GFP cells transfected with WT or R92W *NR5A1::mCherry.* Expression are shown as the ratio between absolute value in SF1GFP+/mCherry+ cells over absolute value in SF1GFP+/mCherry- cells. *Rpl19* was used as reference gene, the values correspond to the mean of two independent experiments.

**Supplemental Tables:**

| Chromosome | Position (hg19) | Shared Haplotype (PAT1) | Second Paternal Haplotype (PAT2) | Second Proband Haplotype (MAT1) |
| --- | --- | --- | --- | --- |
| 9 | 127254221 | T | C | C |
| 9 | 127254230 | C | G | G |
| 9 | 127254350 | C | C | C |
| 9 | 127254523 | T | T | T |
| 9 | 127254857 | T | T | T |
| 9 | 127254859 | C | T | C |
| 9 | 127255441 | A | G | G |
| 9 | 127255448 | G | G | G |
| 9 | 127255526 | G | A | A |
| 9 | 127255614 | G | A | A |
| 9 | 127256223 | T | C | T |
| 9 | 127256995 | A | C | C |
| 9 | 127257557 | C | T | C |
| 9 | 127258486 | A | C | C |
| 9 | 127259048 | A | G | G |
| 9 | 127259472 | G | T | G |
| 9 | 127260640 | G | T | G |
| 9 | 127261893 | G | A | G |
| 9 | 127262043 | A | G | G |
| 9 | 127262864 | C | T | C |
| 9 | 127263719 | A | G | G |
| 9 | 127264512 | G | C | C |
| 9 | 127266504 | G | A | A |
| 9 | 127266648 | G | T | T |
| 9 | 127270068 | T | C | C |
| 9 | 127270499 | G | C | C |

**Supplemental Table 1.** Reconstructed Haplotypes from the Incomplete Trio.

| Individual (ethnicity) | Haplotype | Insertion present? | Variant/total reads | Insertion Phased to Rare Haplotype by Read-Pairs |
| --- | --- | --- | --- | --- |
| HG03135 (ESN) | PAT1 | Yes | 4/8 | No |
| HG03271 (ESN) | PAT1 | Yes | 3/10 | No |
| HG03298 (ESN) | PAT1 | Yes | 4/14 | Yes |
| HG03366 (ESN) | PAT1 | Yes | 3/4 | No |
| HG03517 (ESN) | PAT1 | Yes | 4/13 | No |
| HG02256 (ACB) | PAT1 | Yes | 4/9 | No |
| HG02337 (ACB) | PAT1 | Yes | 4/9 | No |
| HG03054 (MSL) | PAT1 | Yes | 2/11 | Yes |
| HG03069 (MSL) | PAT1 | Yes | 3/4 | Yes |
| HG02562 (GWD) | PAT1 | Yes | 9/14 | No |
| NA19440 (LWK) | PAT1 | Yes | 4/11 | No |
| NA19922 (ASW) | MAT1 | No | 0/11 | NA |
| NA20298 (ASW) | MAT1 | No | 0/18 | NA |
| NA20332 (ASW) | MAT1 | No | 0/6 | NA |
| HG01986 (ACB) | MAT1 | No | 0/7 | NA |
| HG02255 (ACB) | MAT1 | No | 0/6 | NA |
| HG02476 (ACB) | MAT1 | No | 0/7 | NA |
| HG02489 (ACB) | MAT1 | No | 0/8 | NA |
| HG02594 (GWD) | MAT1 | No | 0/14 | NA |
| HG02721 (GWD) | MAT1 | No | 0/8 | NA |
| HG02854 (GWD) | MAT1 | No | 0/8 | NA |
| HG02870 (GWD) | MAT1 | No | 0/3 | NA |
| HG03039 (GWD) | MAT1 | No | 0/9 | NA |
| HG03052 (MSL) | MAT1 | No | 0/13 | NA |
| HG03072 (MSL) | MAT1 | No | 0/10 | NA |
| HG03091 (MSL) | MAT1 | No | 0/4 | NA |
| NA19316 (LWK) | MAT1 | No | 0/3 | NA |
| NA19401 (LWK) | MAT1 | No | 0/25 | NA |
| NA19443 (LWK) | MAT1 | No | 0/15 | NA |
| NA19475 (LWK) | MAT1 | No | 0/6 | NA |

**Supplemental Table 2.** Haplotype Review in 1000 Genomes Data.

**SUPPLEMENTAL REFERENCES**

1. Lourenço, D., Brauner, R., Lin, L., De Perdigo, A., Weryha, G., Muresan, M., Boudjenah, R., Guerra-Junior, G., Maciel-Guerra, A.T., Achermann, J.C., et al. (2009) Mutations in NR5A1 associated with ovarian insufficiency. *N. Engl. J. Med.,* **360**, 1200-1210.
2. Murphy, M.W., Lee, J.K., Rojo, S., Gearhart, M.D., Kurahashi, K., Banerjee, S., Loeuille, G.A., Bashamboo, A., McElreavey, K., Zarkower, D., et al. (2015) An ancient protein-DNA interaction underlying metazoan sex determination. *Nat. Struct. Mol. Biol.,* **22**, 442-451.
3. Chen, K.1., Wallis, J.W., McLellan, M.D., Larson, D.E., Kalicki, J.M., Pohl, C.S., McGrath, S.D., Wendl, M.C., Zhang, Q., Locke, D.P., et al. (2009) BreakDancer: an algorithm for high-resolution mapping of genomic structural variation. *Nat. Methods* **9***,* 677-681.
4. Zeitouni, B.1., Boeva, V., Janoueix-Lerosey, I., Loeillet, S., Legoix-né, P., Nicolas, A., Delattre, O., Barillot, E. (2010) SVDetect: a tool to identify genomic structural variations from paired-end and mate-pair sequencing data. *Bioinformatics* **26**, 1895-1896.
5. MacDonald , J.R., Ziman, R., Yuen, R.K., Feuk, L., Scherer, S.W. (2014) The Database of Genomic Variants: a curated collection of structural variation in the human genome. *Nucleic Acids Res.,* **42**, 986-992.
6. She, X.1., Jiang, Z., Clark, R.A., Liu, G., Cheng, Z., Tuzun, E., Church, D.M., Sutton, G., Halpern, A.L., Eichler, E.E. (2004) Shotgun sequence assembly and recent segmental duplications within the human genome. *Nature* **431**, 927-930.
7. Fiume, M.1., Smith, E.J., Brook, A., Strbenac, D,. Turner, B., Mezlini, A.M., Robinson, M.D., Wodak, S.J., Brudno, M. (2012) Savant Genome Browser 2: visualization and analysis for population-scale genomics. *Nucleic Acids Res.,* **40**, 615-621.
8. Rimmer, A., Phan, H.1., Mathieson, I., Iqbal, Z., Twigg, S.R.,WGS500 Consortium, Wilkie, A.O., McVean, G., Lunter, G. (2014) Integrating mapping-, assembly- and haplotype-based approaches for calling variants in clinical sequencing applications. *Nat. Genet.,* **46**, 912-918.
9. Reid, J.G., Carroll, A., Veeraraghavan, N., Dahdouli, M., Sundquist, A,. English, A., Bainbridge, M., White, S., Salerno, W., Buhay, C., et al. (2014) Launching genomics into the cloud: deployment of Mercury, a next generation sequence analysis pipeline. *BMC Bioinformatics* **15***,* 30.
10. Abecasis, G.R., Altshuler, D., Auton, A., Brooks, L.D., Durbin, R.M., Gibbs, R.A., Hurles, M.E., McVean, G.A., 1000 Genomes Project Consortium (2010) A map of human genome variation from population-scale sequencing. *Nature* **467***,* 1061-1073.
11. Sali, A., and Blundell, T.L. (1993) Comparative protein modelling by satisfaction of spatial restraints. *J. Mol. Biol.,* **234**, 779-815.
12. Blind, R.D., Sablin, E.P., Kuchenbecker, K.M., Chiu, H.J., Deacon, A.M., Das, D., Fletterick, R.J., Ingraham, H.A. (2014) The signaling phospholipid pip3 creates a new interaction surface on the nuclear receptor sf-1. *Proc. Natl. Acad. Sci. U. S. A.,* **111**, 15054-15059.
13. Sablin, E.P., Woods, A., Krylova, I.N., Hwang, P., Ingraham, H.A., and Fletterick, R.J. (2008) The structure of corepressor dax-1 bound to its target nuclear receptor lrh-1. *Proc. Natl. Acad. Sci. U. S. A.,* **105**, 18390-18395.
14. Li, Y., Choi, M., Cavey, G., Daugherty J, Suino, K., Kovach, A., Bingham, N.C., Kliewer, S.A., and Xu, H.E. (2005) Crystallographic identification and functional characterization of phospholipids as ligands for the orphan nuclear receptor steroidogenic factor-1. *Mol. Cell* **17**, 491-502.
15. Little, T.H., Zhang, Y., Matulis, C.K., Weck, J., Zhang, Z., Ramachandran, A., Mayo, K.E., and Radhakrishnan, I. (2006) Sequence-specific deoxyribonucleic acid (DNA) recognition by steroidogenic factor 1: A helix at the carboxy terminus of the DNA binding domain is necessary for complex stability. *Mol. Endocrinol.,* **20**, 831-843.
16. Brooks, B.R., Brooks, C.L., Mackerell, A.D., Nilsson, L., Petrella, R.J., Roux, B., Won, Y., Archontis, G., Bartels, C., Boresch, S., et al. (2009) Charmm: The biomolecular simulation program. *J. Comput. Chem.,* **30**, 1545-1614.
17. Lovell, S.C., Davis, I.W., Arendall, W.B., de Bakker, P.I.W., Word, J.M., Prisant, M.G., Richardson, J.S., and Richardson, D.C. (2003) Structure validation by cα geometry: Φ,ψ and cβ deviation. *Proteins: Structure, Function, and Bioinformatics* **50**, 437-450
18. Wiederstein, M., and Sippl, M.J. (2007) Prosa-web: Interactive web service for the recognition of errors in three-dimensional structures of proteins. *Nucl. Acid. Res*., **35**, W407-W410.
19. Dolinsky, T.J., Czodrowski, P., Li, H., Nielsen, J.E., Jensen J,H., Klebe, G., Baker, N.A. (2007) Pdb2pqr: Expanding and upgrading automated preparation of biomolecular structures for molecular simulations. *Nucl. Acid. Res.,* **35**, W522-W525.
20. Eisenberg, D., Schwarz, E., Komaromy, M., and Wall, R. (1984) Analysis of membrane and surface protein sequences with the hydrophobic moment plot. *J. Mol. Biol.,* **179**, 125-142.
21. Korinek, V., Barker, N., Morin, P.J., van Wichen, D., de Weger, R., Kinzler, K.W., Vogelstein, B., and Clevers, H. (1997) Constitutive transcriptional activation by a beta-catenin-Tcf complex in APC-/- colon carcinoma. *Science.,* **275**, 1784–1787.
22. Mizusaki, H., Kawabe, K., Mukai, T., Ariyoshi, E., Kasahara, M., Yoshioka, H., Swain, A., and Morohashi, K. (2003) Dax-1 (dosage-sensitive sex reversal-adrenal hypoplasia congenita critical region on the X chromosome, gene 1) gene transcription is regulated by wnt4 in the female developing gonad*. Mol. Endocrinol.,* **17**, 507-519.
23. Stallings, N.R., Hanley, N.A., Majdic, G., Zhao, L., Bakke, M., and Parker K.L. (2002) Development of a transgenic green fluorescent protein lineage marker for steroidogenic factor 1. *Mol. Endocrinol.,* **16**, 2360-2370.
24. Pitetti, J.L., Calvel, P., Zimmermann, C., Conne, B., Papaioannou, M.D., Aubry, F., Cederroth, C.R., Urner, F., Fumel, B., Crausaz, M., et al., (2013) An essential role for insulin and IGF1 receptors in regulating sertoli cell proliferation, testis size, and FSH action in mice. *Mol. Endocrinol.,* **27**, 814-827.
25. Sudmant, P.H., Rausch, T., Gardner, E.J., Handsaker, R.E., Abyzov, A., Huddleston, J., Zhang, Y., Ye, K., Jun, G., Hsi-Yang Fritz, M., et al. (2015) An integrated map of structural variation in 2,504 human genomes. *Nature.,* **526**, 75-81.
26. Thorvaldsdóttir, H., Robinson, J.T., and Mesirov, J.P. Integrative Genomics Viewer (IGV): high-performance genomics data visualization and exploration. *Brief. Bioinform.,* **14**, 178-192.
