# Supplementary material for: A recurrent p.Arg92Trp variant in steroidogenic factor-1 (NR5A1) can act as a molecular switch in human sex development
Source: Hum Mol Genet. 2016 Jul 4;25(16):3446–53. doi: 10.1093/hmg/ddw186 (PMC5179941; doi:10.1093/hmg/ddw186)
Supplement: Supplementary Data [file supp_ddw186_Supplemental_UDN_Member_list.pdf]

First	Last	Affiliation	Email	Role in the UDN (ex. PI, site coordinator)
Carlos	Bacino	BCM Clinical	cbacino@bcm.edu	Lead clinician, pediatrics
Brendan	Lee	BCM Clinical	blee@bcm.edu	PI
Ashok	Balasubramanyam	BCM Clinical	ashokb@bcm.edu.	Lead clinician, adult
Lindsay	Burrage	BCM Clinical	burrage@bcm.edu	Sequence analysis team, pediatric genetics
Gary	Clark	BCM Clinical	gdclark@texaschildrens.org	Pediatric neurology
William	Craigen	BCM Clinical	wcraigen@bcm.edu	Leadership team, pediatric genetics, biochemical genetics
Shweta	Dhar	BCM Clinical	dhar@bcm.edu	Adult genetics
Lisa	Emrick	BCM Clinical	emrick@bcm.edu	Leadership team, pediatric neurology
Brett	Graham	BCM Clinical	bgraham@bcm.edu	Pediatric Genetics, Biochemical Genetics, Mitochondrial function core
Mahim	Jain	BCM Clinical	mahimj@bcm.edu	Sequence analysis team, Pediatric Genetics
Seema	Lalani	BCM Clinical	seemal@bcm.edu	Pediatric genetics, Cytogenetics
Richard	Lewis	BCM Clinical	rlewis@bcm.edu	Ophthalmology
Paolo	Moretti	BCM Clinical	pmoretti@bcm.edu	Leadership team, adult neurology
Sarah	Nicholas	BCM Clinical	sknichol@bcm.edu	Allergy & Immunology
Jordan	Orange	BCM Clinical	orange@bcm.edu	Leadership team, allergy & Immunology
Jennifer	Posey	BCM Clinical	Jennifer.Posey@bcm.edu	Adult genetics
Lorraine	Potocki	BCM Clinical	lpotocki@bcm.edu	Pediatric genetics
Jill	Rosenfeld	BCM Clinical	mokry@bcm.edu	Site Manager
Daryl	Scott	BCM Clinical	dscott@bcm.edu	Pediatric genetics
Neil	Hanchard	BCM Clinical	hanchard@bcm.edu	Pediatric Genetics & Genomics Core
Tran	Alyssa	BCM Clinical	alyssat@bcm.edu	Site coordinator
Alejandro	Mercedes	BCM Clinical	mercedes@bcm.edu	Site coordinator
Azamian	Mashid	BCM Clinical	azamian@bcm.edu	Site coordinator, clinical assistant
Hugo	Bellen	BCM MOSC	hbellen@bcm.edu	PI
Christine	Eng	BCM Sequencing	ceng@bcm.edu	PI, Molecular Geneticist
Yaping	Yang	BCM Sequencing	yapingy@bcm.edu	Laboratory Director, Molecular Geneticist
Donna	Muzny	BCM Sequencing	donnam@bcm.edu	Director of Operations BCM-HGSC
Patricia	Ward	BCM Sequencing	pward@bcm.edu	Site Coordinator and Genetic Counselor
Rachel	Ramoni	Harvard CC	rachel_ramoni@hms.harvard.edu	PI
Alexa	McCray	Harvard CC	alexa_mccray@hms.harvard.edu	PI
Issac	Kohane	Harvard CC	isaac_kohane@hms.harvard.edu	PI
Ingrid	Holm	Harvard CC	ingrid.holm@childrens.harvard.edu	Associate professor
Matthew	Might	Harvard CC	matthew_might@hms.harvard.edu	Patient Advisor
Paul	Mazur	Harvard CC	paul_mazur@hms.harvard.edu	Project Coordinator
Kimberly	Splinter	Harvard CC	kimberly_splinter@hms.harvard.edu	Genetic Counselor and Clinical Project Manager
Cecilia	Esteves	Harvard CC	cecilia_esteves@hms.harvard.edu	Sequencing Project Manager
Vandana	Shashi	Duke	vandana.shashi@duke.edu	PI
Yong-Hui	Jiang	Duke	yong-hui.jiang@dm.duke.edu	Clinician
Loren	Del Mar Pena	Duke	loren.pena@dm.duke.edu	Clinician
Allyn	McConkie-Rosell	Duke	allyn.mcconkie@duke.edu	Genetic Counselor
Kelly	Schoch	Duke	kelly.schoch@duke.edu	Genetic Counselor

Rebecca	Spillman	Duke	rebecca.crimian@duke.edu	Genetic Counselor
Jennifer	Sullivan	Duke	jennifer.sullivan@duke.edu	Genetic Counselor
Nicole	Walley	Duke	nicole.walley@duke.edu	Clinical Research Coordinator
David	Goldstein	Columbia	dg2875@columbia.edu	PI
Nicholas	Stong	Columbia	ns3116@cumc.columbia.edu	Bioinformatician
Alan	Beggs	Harvard	beggs@enders.tch.harvard.edu	Site PI
Joseph	Loscalzo	Harvard	jloscalzo@partners.org	PI
Calum	MacRae	Harvard	camacrae@bics.bwh.harvard.edu	Site PI
Edwin	Silverman	Harvard	ed.silverman@channing.harvard.edu	Site PI
Joan	Stoler	Harvard	joan.stoler@childrens.harvard.edu	Site PI
David	Sweetser	Harvard	dsweetser@partners.org	Site PI
Richard	Maas	Harvard	maas@genetics.med.harvard.edu	Site PI
Joel	Krier	Harvard	jkrier@partners.org	Site PI
Lance	Rodan	Harvard	lance.rodan@childrens.harvard.edu	UDN Fellow
Chris	Walsh	Harvard	Christopher.Walsh@childrens.harvard.e	Site PI
Cynthia	Cooper	Harvard	CCOOPER1@mgh.harvard.ed	Site PI
Carl	Pallais	Harvard	Juan.Pallais@mgh.harvard.edu	Site PI
Howard	Jacob	HudsonAlpha	hjacob@hudsonalpha.org	PI
Elizabeth	Worthey	HudsonAlpha	lworthey@hudsonalpha.org	PI and Informatics Director
Joe	Lazar	HudsonAlpha	jlazar@hudsonalpha.org	Site coordinator
Kim	Strong	HudsonAlpha	kstrong@hudsonalpha.org	Bioethicist and Director, Ethics and Genomics Program
Lori	Handley	HudsonAlpha	lhandley@hudsonalpha.org	Project Manager
Scott	Newberry	HudsonAlpha	snewberry@hudsonalpha.org	Software Architect
David	Bick	HudsonAlpha	dbick@hudsonalpha.org	Clinical Molecular Geneticist
Molly	Schroeder	HudsonAlpha	mschroeder@hudsonalpha.org	Clinical Molecular Geneticist
Donna	Brown	HudsonAlpha	dbrown@clinicallab.org	Clinical Analyst
Camille	Birch	HudsonAlpha	cbirch@clinicallab.org	Clinical Analyst
Shawn	Levy	HudsonAlpha	slevy@hudsonalpha.org	Director, Clinical Sequencing Lab (CSL)
Braden	Boone	HudsonAlpha	bboone@hudsonalpha.org	CSL Senior Scientist
Dan	Dorset	HudsonAlpha	ddorset@hudsonalpha.org	CSL Computational Biologist
Angela	Jones	HudsonAlpha	ajones@hudsonalpha.org	CSL Senior Scientist
Teri	Manolio	NIH	manolio@nih.gov	Program Director
John	Mulvihill	NIH	John.Mulvihill@nih.gov	Senior Consultant
Anastasia	Wise	NIH	anastasia.wise@nih.gov	Program Director
Jyoti	Dayal	NIH	jyotig@mail.nih.gov	Program Specialist
David	Eckstein	NIH	david.eckstein@nih.gov	Senior Health Scientist Administrator
Brenda	Iglesias	NIH	brenda.iglesias@nih.gov	Scientific Program Analyst
Donna	Krasnewich	NIH	dkras@mail.nih.gov	Program Officer
Carson	Loomis	NIH	loomisc@mail.nih.gov	Program Director
Laura	Mamounas	NIH	mamounas@ninds.nih.gov	Program Director
Casey	Martin	NIH	casey.martin@nih.gov	Scientific Program Analyst
David	Koeller	OHSU Metabolomics	koellerd@ohsu.edu	PI

Thomas	Metz	PNNL Metabolomics	thomas.metz@pnnl.gov	PI
Euan	Ashley	Stanford	Euan@stanford.edu	PI
Paul	Fisher	Stanford	pfisher@stanford.edu	PI
Jonathan	Bernstein	Stanford	Jon.Bernstein@stanford.edu	PI
Matt	Wheeler	Stanford	wheelerm@stanford.edu	Executive & Medical Director, Adults
Patricia	Zornio	Stanford	pzornio@stanford.edu	Lead Study Coordinator
Daryl	Waggott	Stanford	dwaggott@stanford.edu	Bioinformatician
Annika	Dries	Stanford	annikamd@stanford.edu	Study Coordinator
Jennefer	Kohler	Stanford	jkohler@stanfordhealthcare.org	Genetic Counselor
Katrina	Dipple	UCLA	Kdipple@mednet.ucla.edu	PI
Stan	Nelson	UCLA	snelson@ucla.edu	PI
Christina	Palmer	UCLA	cpalmer@mednet.ucla.edu	PI
Eric	Vilain	UCLA	evilain@ucla.edu	PI
Patrick	Allard	UCLA	pallard@ucla.edu	Co-investigator
Esteban	Dell Angelica	UCLA	edellangelica@mednet.ucla.edu	Co-investigator
Hane	Lee	UCLA	hanelee@mednet.ucla.edu	Sequencing interpretation
Janet	Sinsheimer	UCLA	janet@mednet.ucla.edu	Co-investigator
Jeanette	Papp	UCLA	jcpapp@mednet.ucla.edu	Director of the UCLA Genotyping and Sequencing Core
Naghmeh	Dorrani	UCLA	ndorrani@mednet.ucla.edu	Site coordinator and genetic counselor
Matthew	Herzog	UCLA	mherzog@mednet.ucla.edu	Site coordinator and genetic counselor
Hayk	Barseghyan	UCLA	haykbarseghyan@ucla.edu	Graduate student
David	Adams	UDP, NHGRI	david.adams@nih.gov	Deputy Director for Clinical Genomics, OCD/NHGRI
Christopher	Adams	UDP	christopher.adams@nih.gov	Fellow
Elizabeth	Burke	UDP	elizabeth.burke2@nih.gov	Postdoctoral Fellow
Katherine	Chao	UDP	katherine.chao@nih.gov	Fellow
Mariska	Davids	UDP	mariska.davids@nih.gov	Post doctoral fellow
David	Draper	UDP	draperd@mail.nih.gov	Nurse Specialist, Research
Tyra	Estwick	UDP	tyra.estwick@nih.gov	Research Nurse Coordinator
Trevor	Frisby	UDP	trevor.frisby@nih.gov	Fellow
Kate	Frost	UDP	kate.frost@nih.gov	Fellow
William	Gahl	UDP, NHGRI	gahlw@mail.nih.gov	Clinical Director NHGRI
Valerie	Gartner	UDP	valerie.gartner@nih.gov	Fellow
Rena	Godfrey	UDP	godfreyra@mail.nih.gov	Physicians Assistant
Mitchel	Goheen	UDP	mitchell.goheen@nih.gov	Fellow
Gretchen	Golas	UDP	gretchen.golas@nih.gov	Nurse Practitioner
Mary "Gracie"	Gordon	UDP	gracie.gordon@nih.gov	Fellow
Catherine	Groden	UDP	catherine.groden@nih.gov	Nurse Practitioner
Andrea	Gropman	CNMC	agropman@cnmc.org	Staff Clinician
Mary	Hackbarth	UDP	mary.hackbarth@nih.gov	Fellow
Isabel	Hardee	UDP	isabel.hardee@nih.gov	Fellow
Jean	Johnston	UDP	johnstonjm@mail.nih.gov	Research Nurse Coordinator
Alanna	Koehler	UDP	alanna.koehler@nih.gov	Fellow

Lea	Latham	UDP	lea.latham@nih.gov	Nurse Practitioner
Yvonne	Latour	UDP	yvonne.latour@nih.gov	Fellow
Christopher	Lau	UDP	christopher.lau@nih.gov	Molecular Geneticist/CLIA Lab Director
Paul	Lee	UDP, NINDS	paul.lee@nih.gov	Staff Clinician
Denise	Levy	UDP	denise.levy@nih.gov	Fellow
Adam	Liebendorder	UDP	adam.liebendorfer@nih.gov	Fellow
Ellen	Macnamara	UDP	ellen.macnamara@nih.gov	Genetic Counselor
Valerie	Maduro	UDP	vbraden@mail.nih.gov	UDP Translational Laboratory Manager
May	Malicdan	UDP, NHGRI	maychristine.malicdan@nih.gov	Stff Scientist
Thomas	Markello	UDP	markellot@mail.nih.gov	Staff Clinician
Alexandra	McCarty	UDP	alexandra.mccarty@nih.gov	Fellow
Jennifer	Murphy	UDP	jennifer.murphy@nih.gov	Nurse Practitioner
Michele	Nehrebecky	UDP	michele.nehrebecky@nih.gov	Nurse Practitioner
Donna	Novacic	UDP	donna.novacic@nih.gov	Staff Clinician
Barbara	Pusey	UDP	barbara.pusey@nih.gov	Informatician
Sarah	Sadozai	UDP	sarah.sadozai@nih.gov	Fellow
Katherine	Schaffer	UDP	katherine.schaffer@nih.gov	Fellow
Prashant	Sharma	UDP	prashant.sharma@nih.gov	Post-Docotoral Fellow
Ariane	Soldatos	NINDS	ariane.soldatos@nih.gov	Staff Clinician
Sara	Thomas	UDP	sarah.thomas@nih.gov	Fellow
Cynthia	Tifft	UDP, NHGRI	ctifft@nih.gov	Deputy Clinical Director NHGRI
Nate	Tolman	UDP	nate.tolman@nih.gov	Fellow
Camilo	Toro	UDP	toroc@mail.nih.gov	Staff Clinician
Zaheer	Valivullah	UDP	zaheer.valivullah@nih.gov	Contractor/Informaticist
Colleen	Wahl	UDP	colleen.wahl@nih.gov	Nurse Practitioner
Mike	Warburton	UDP	mike.warburton@nih.gov	Fellow
Alec	Weech	UDP	alec.weech@nih.gov	Fellow
Lynne	Wolfe	UDP, NHGRI	lynne.wolfe@nih.gov	Senior Nurse Practitioner
Guoyun	Yu	UDP	guoyun.yu@nih.gov	Head of Informatics, UDP
Rizwan	Hamid	Vanderbilt	rizwan.hamid@vanderbilt.edu	PI
John	Newman	Vanderbilt	john.newman@vanderbilt.edu	PI
John A.	Phillips	Vanderbilt	John.a.phillips@vanderbilt.edu	PI
Joy	Cogan	Vanderbilt	joy.cogan@vanderbilt.edu	Director, Central Biorepository

Institutional Abbreviations
BCM- Baylor College of Medicine & Texas Children's Hospital, Houston, Texas
Harvard- Harvard Medical School & Harvard Teaching Hospitals
UDP- National Institutes of Health
Stanford- Stanford Medicine
UCLA- UCLA School of Medicine
Vanderbilt - Vanderbilty University Medical Center
Hudson Alpha Institute for Biotechnology
PNNL Metabolomics - Pacific Northwest National Laboratories

OHSU Metabolomics - Oregon Health Sciences University
Duke - Duke Medicine
Colubmia - Columbia University
MOSC - Model Organisms Screening Center - BCM and University of Oregon
