# Supplementary figures and images for: A recurrent p.Arg92Trp variant in steroidogenic factor-1 (NR5A1) can act as a molecular switch in human sex development
Source: Hum Mol Genet. 2016 Jul 4;25(16):3446–53. doi: 10.1093/hmg/ddw186 (PMC5179941; doi:10.1093/hmg/ddw186)

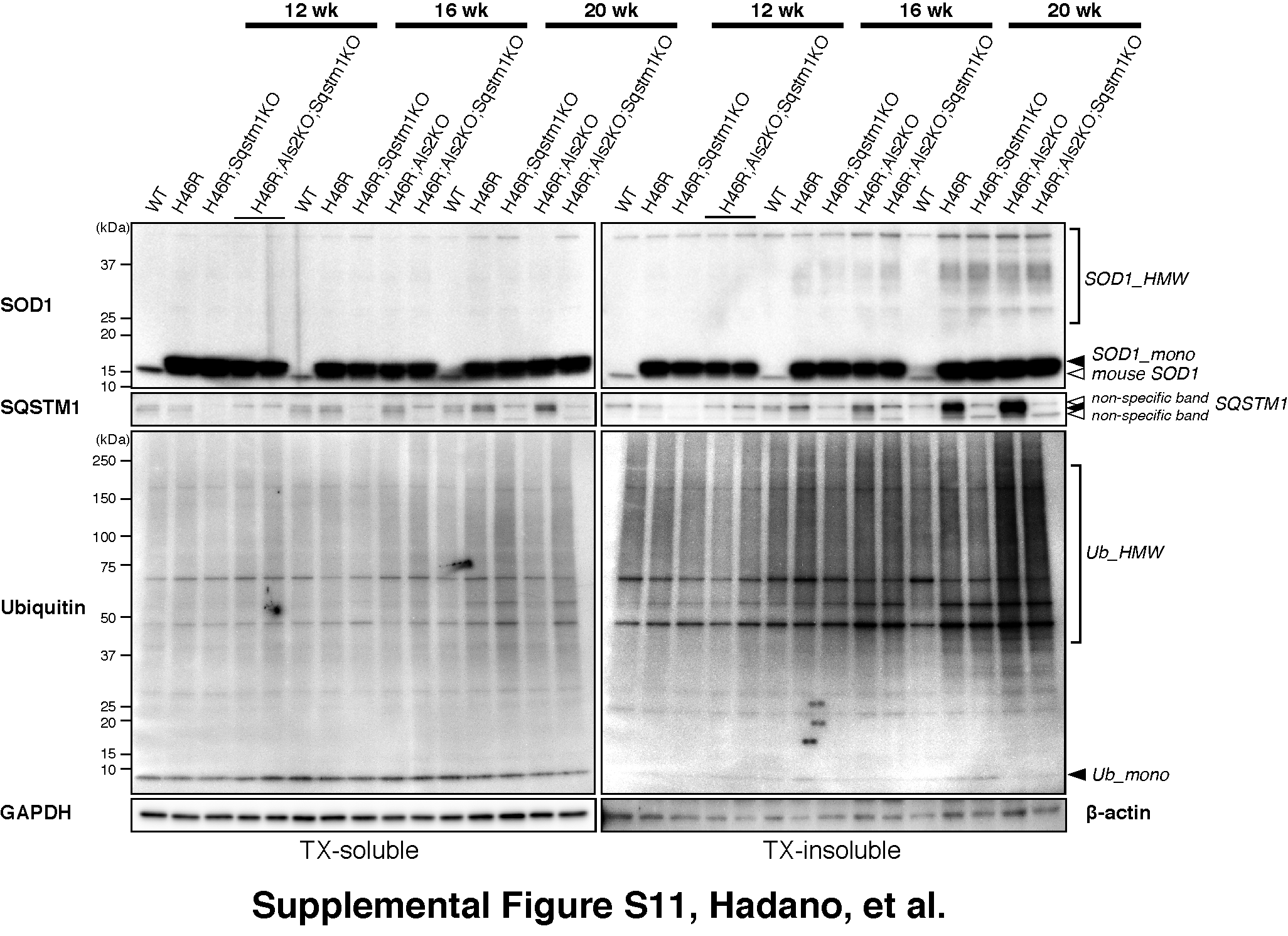

Supplement: Supplementary Data [file supp_ddw186_suppl_data.zip › FigS11.tif]

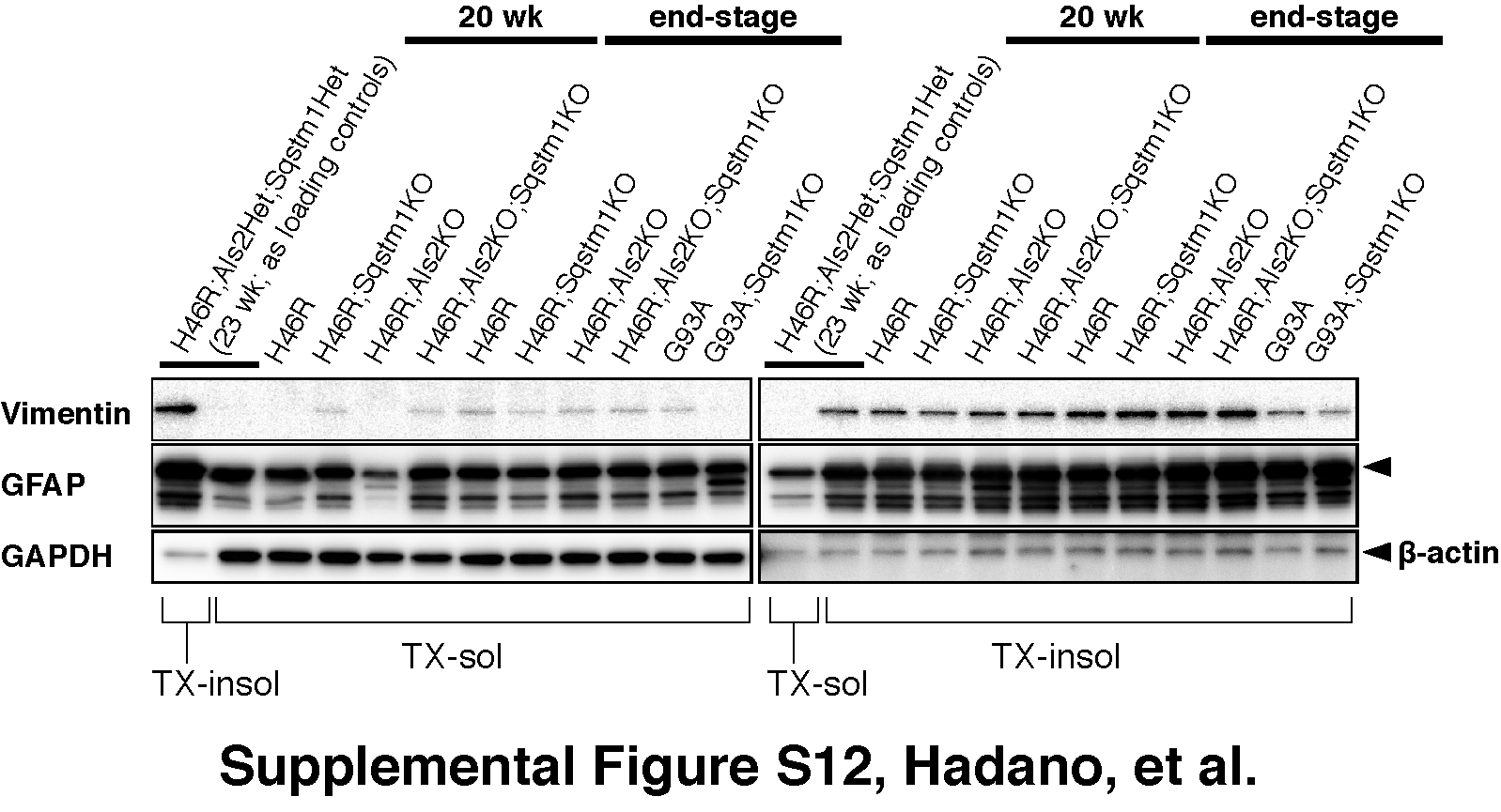

Supplement: Supplementary Data [file supp_ddw186_suppl_data.zip › FigS12.tif]

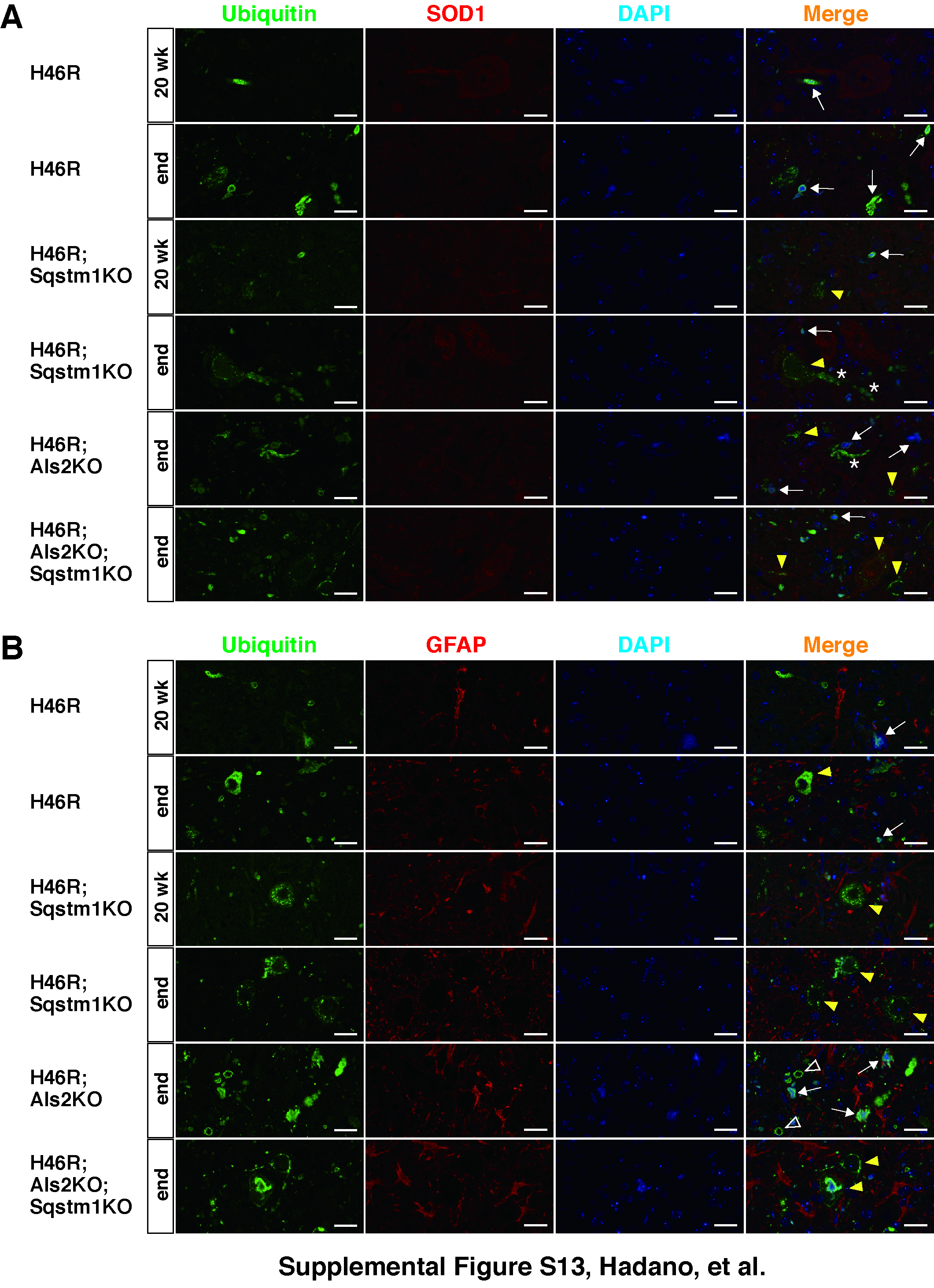

Supplement: Supplementary Data [file supp_ddw186_suppl_data.zip › FigS13.tif]

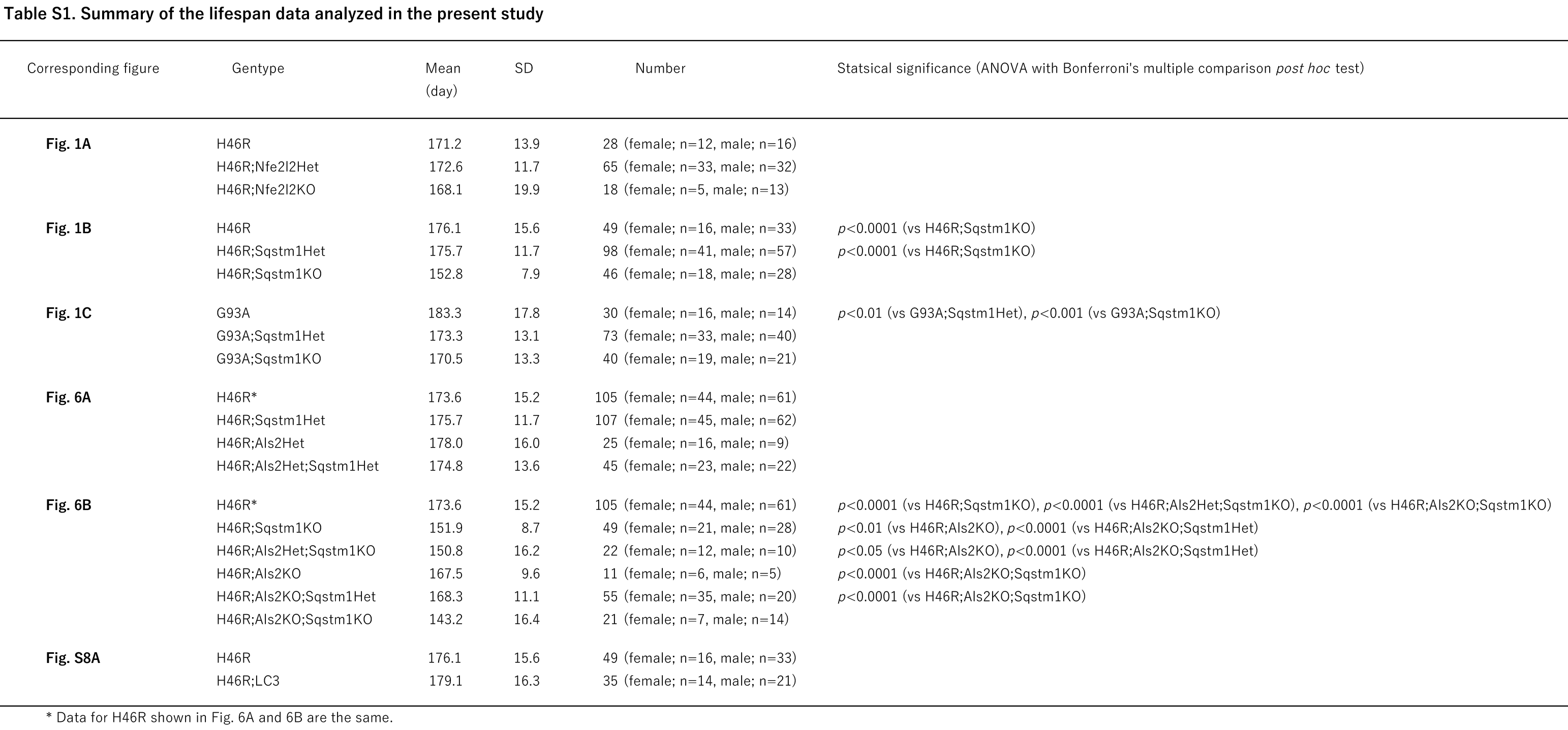

Supplement: Supplementary Data [file supp_ddw186_suppl_data.zip › TableS1v2.tif]

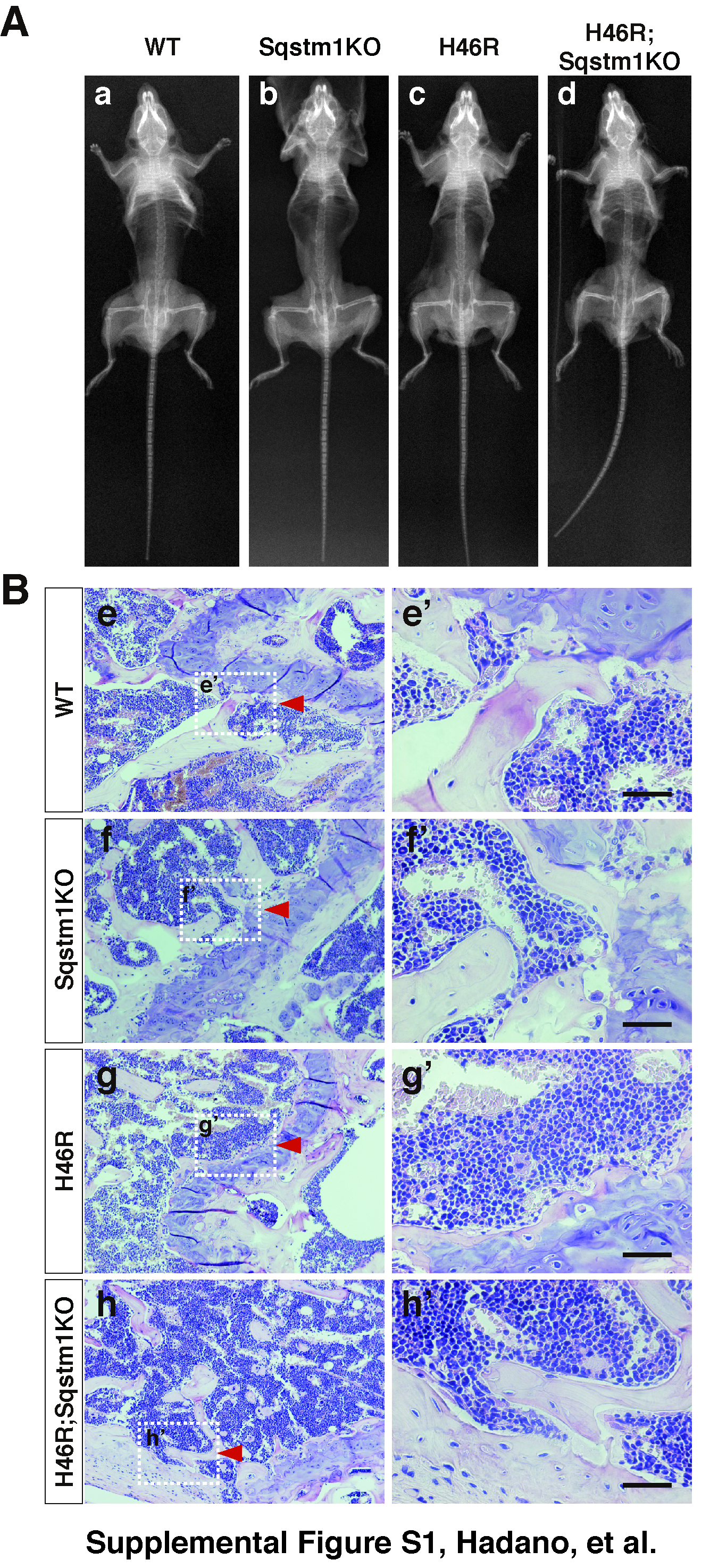

Supplement: Supplementary Data [file supp_ddw186_suppl_data.zip › FigS1.tif]

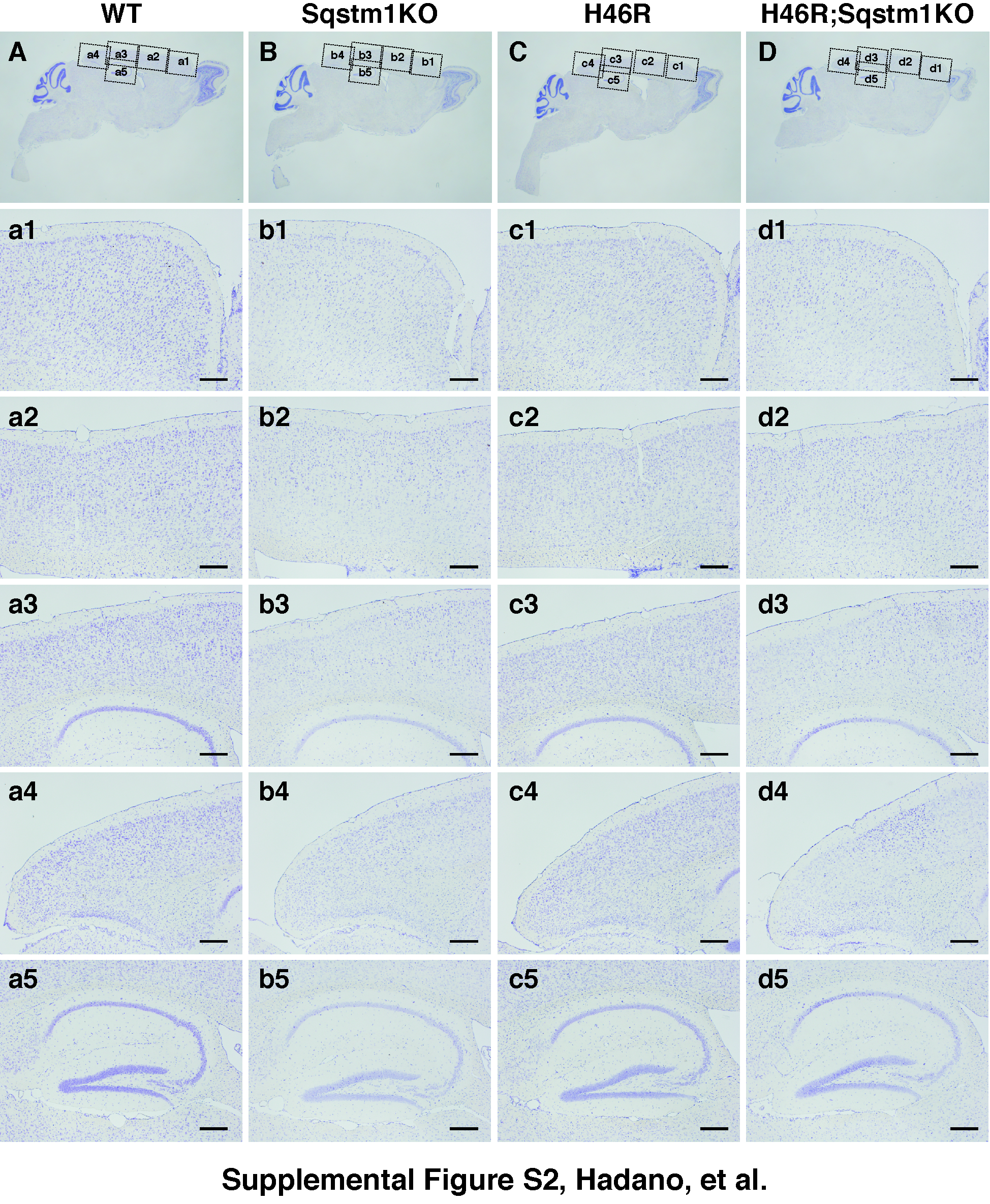

Supplement: Supplementary Data [file supp_ddw186_suppl_data.zip › FigS2.tif]

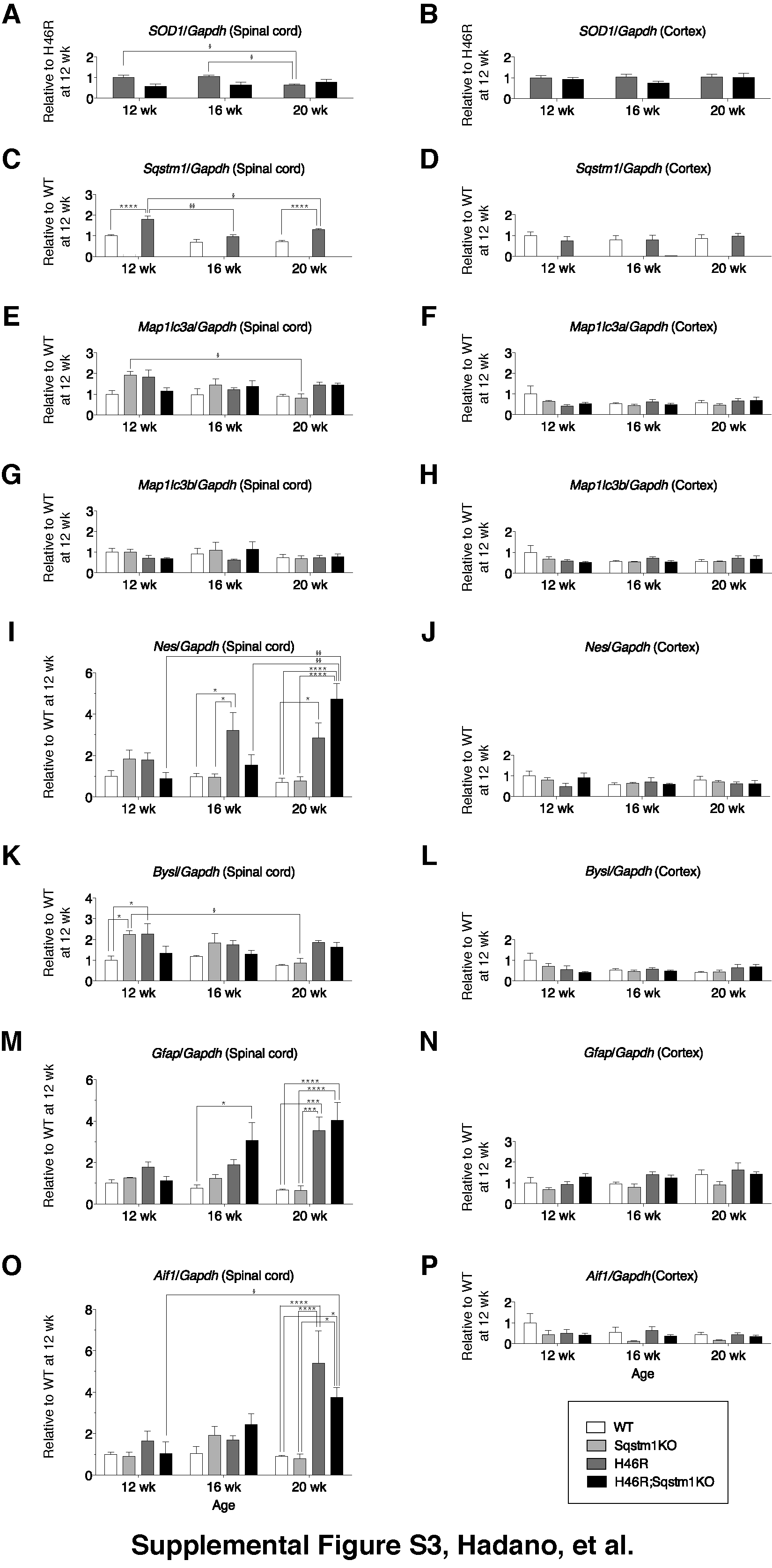

Supplement: Supplementary Data [file supp_ddw186_suppl_data.zip › FigS3.tif]

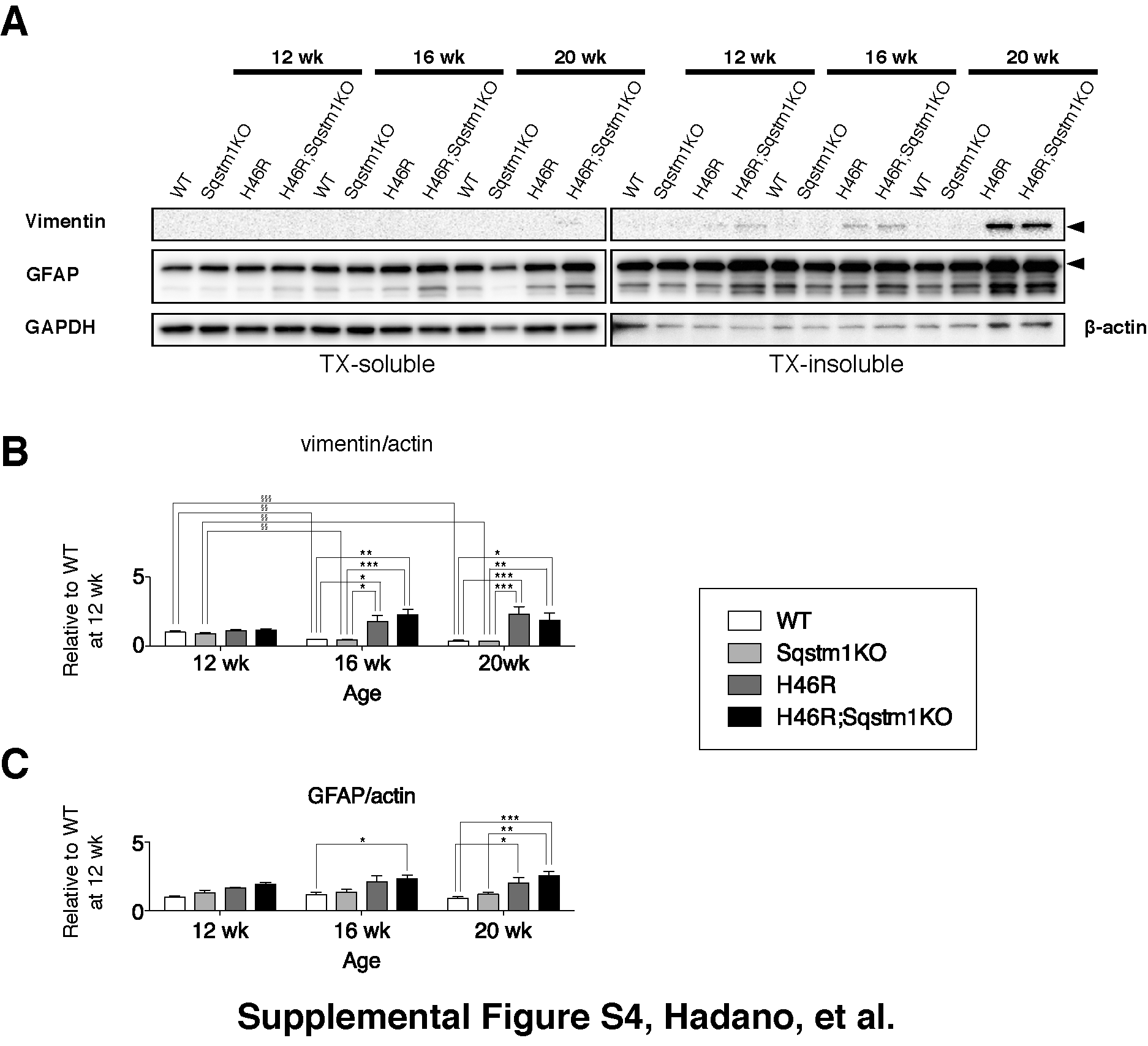

Supplement: Supplementary Data [file supp_ddw186_suppl_data.zip › FigS4.tif]

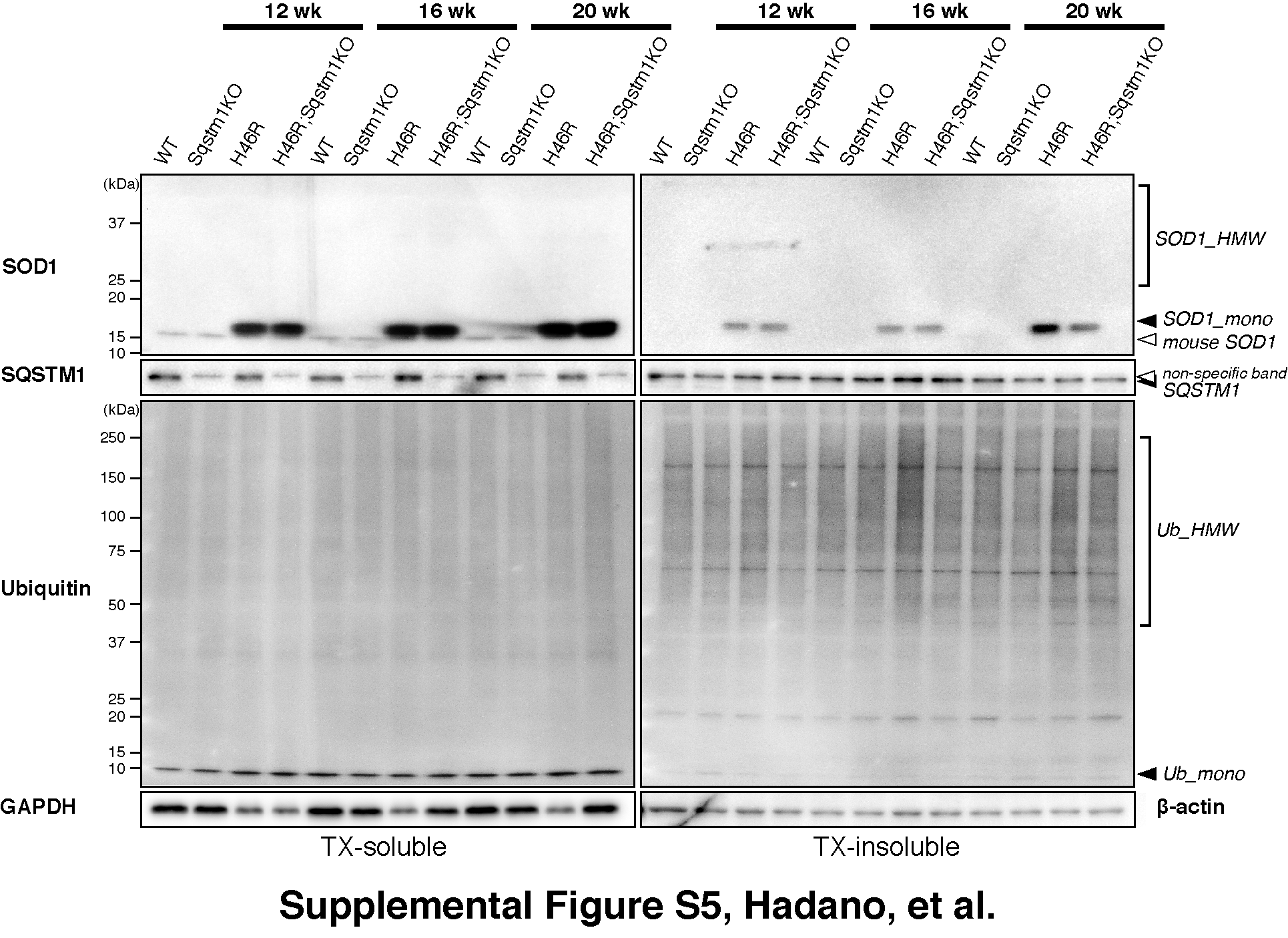

Supplement: Supplementary Data [file supp_ddw186_suppl_data.zip › FigS5.tif]

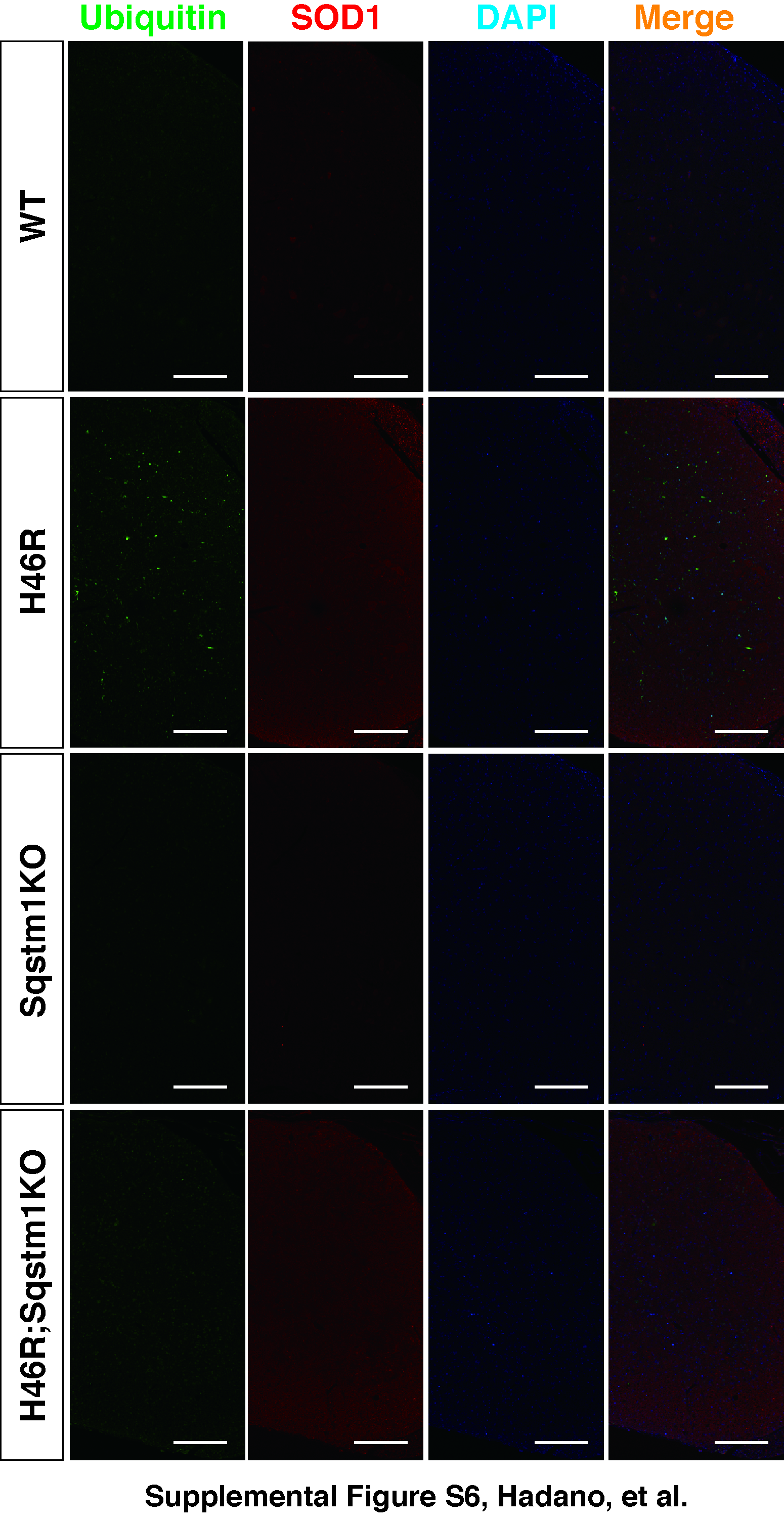

Supplement: Supplementary Data [file supp_ddw186_suppl_data.zip › FigS6.tif]

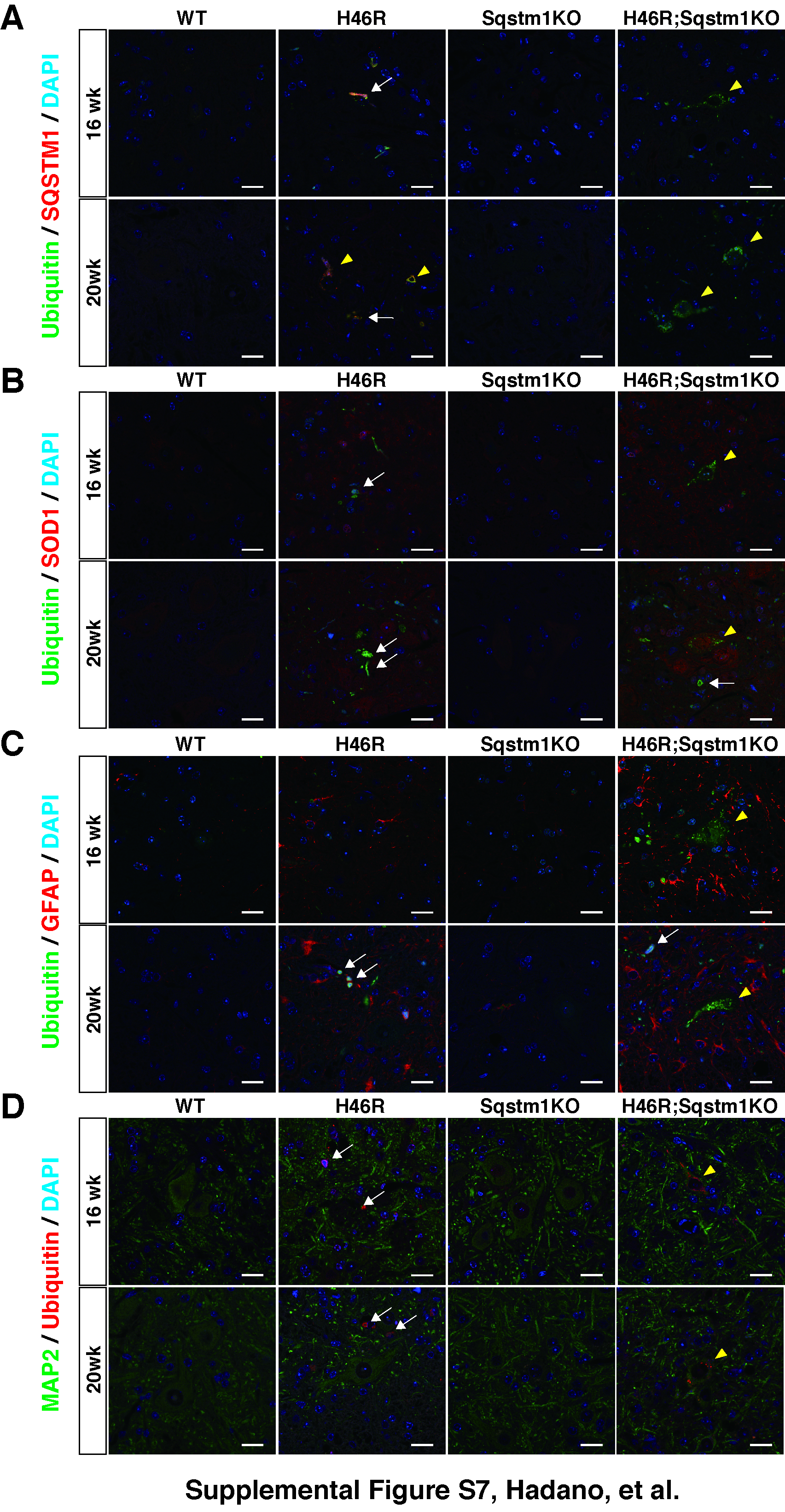

Supplement: Supplementary Data [file supp_ddw186_suppl_data.zip › FigS7.tif]

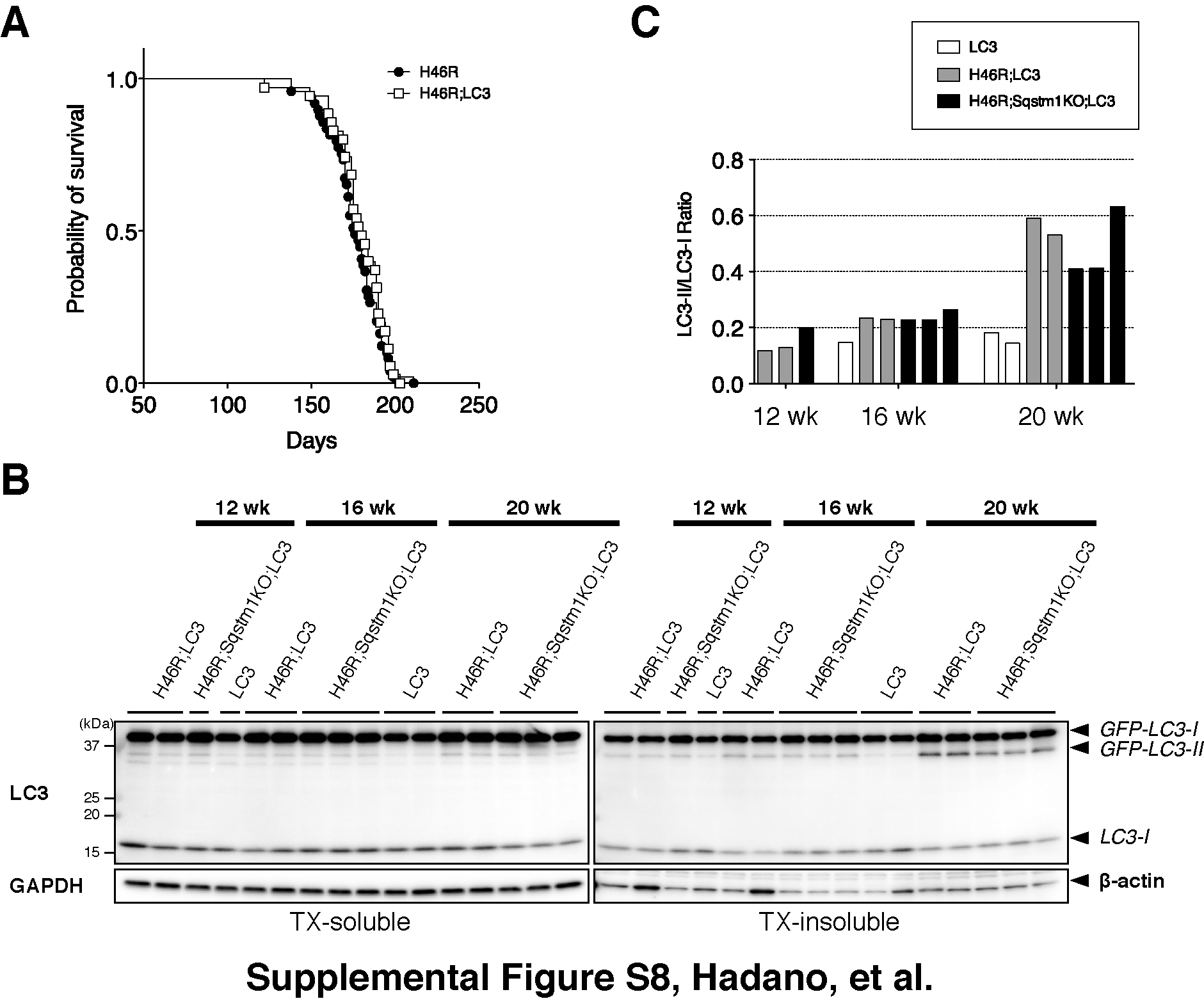

Supplement: Supplementary Data [file supp_ddw186_suppl_data.zip › FigS8.tif]

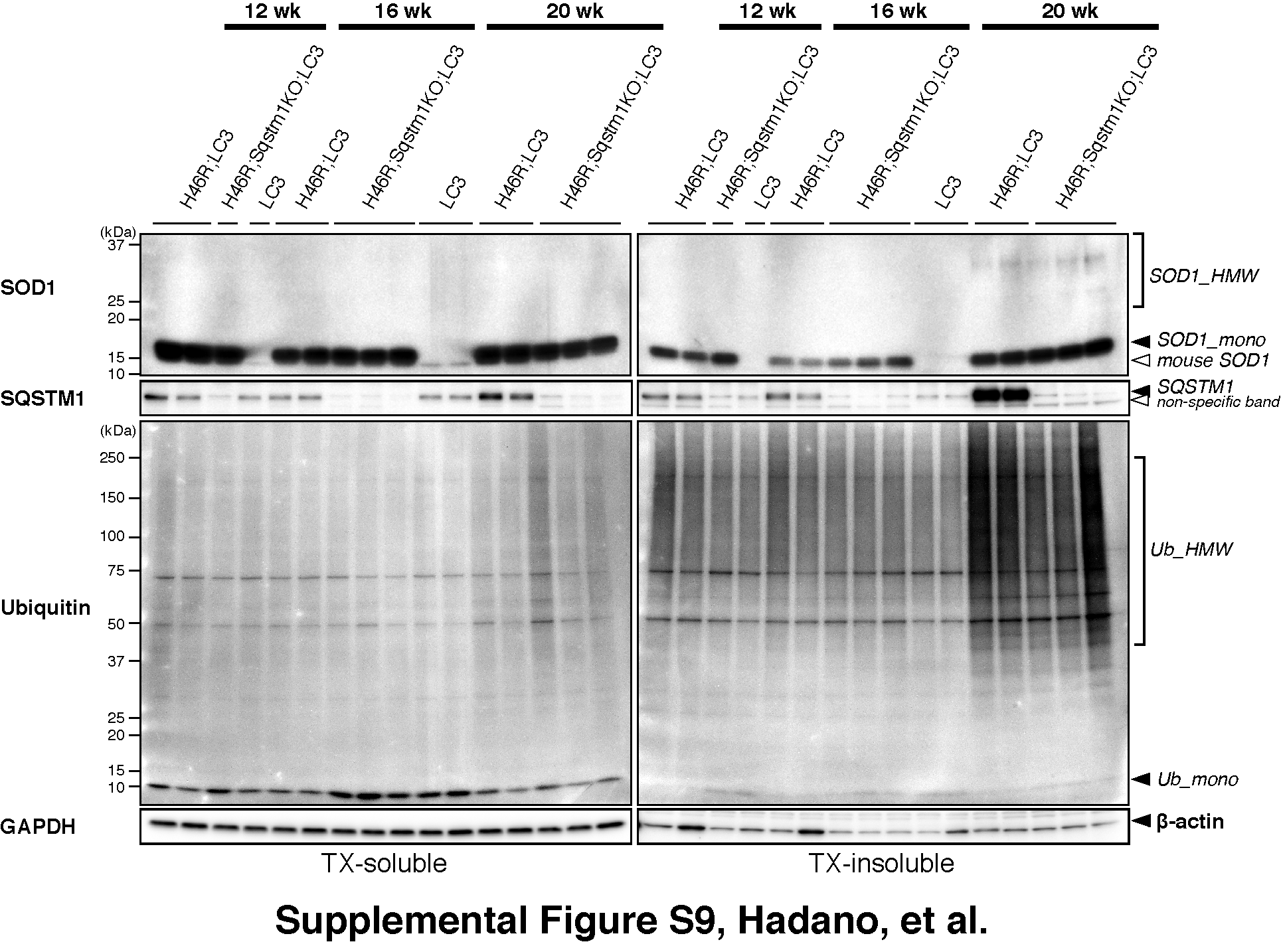

Supplement: Supplementary Data [file supp_ddw186_suppl_data.zip › FigS9.tif]

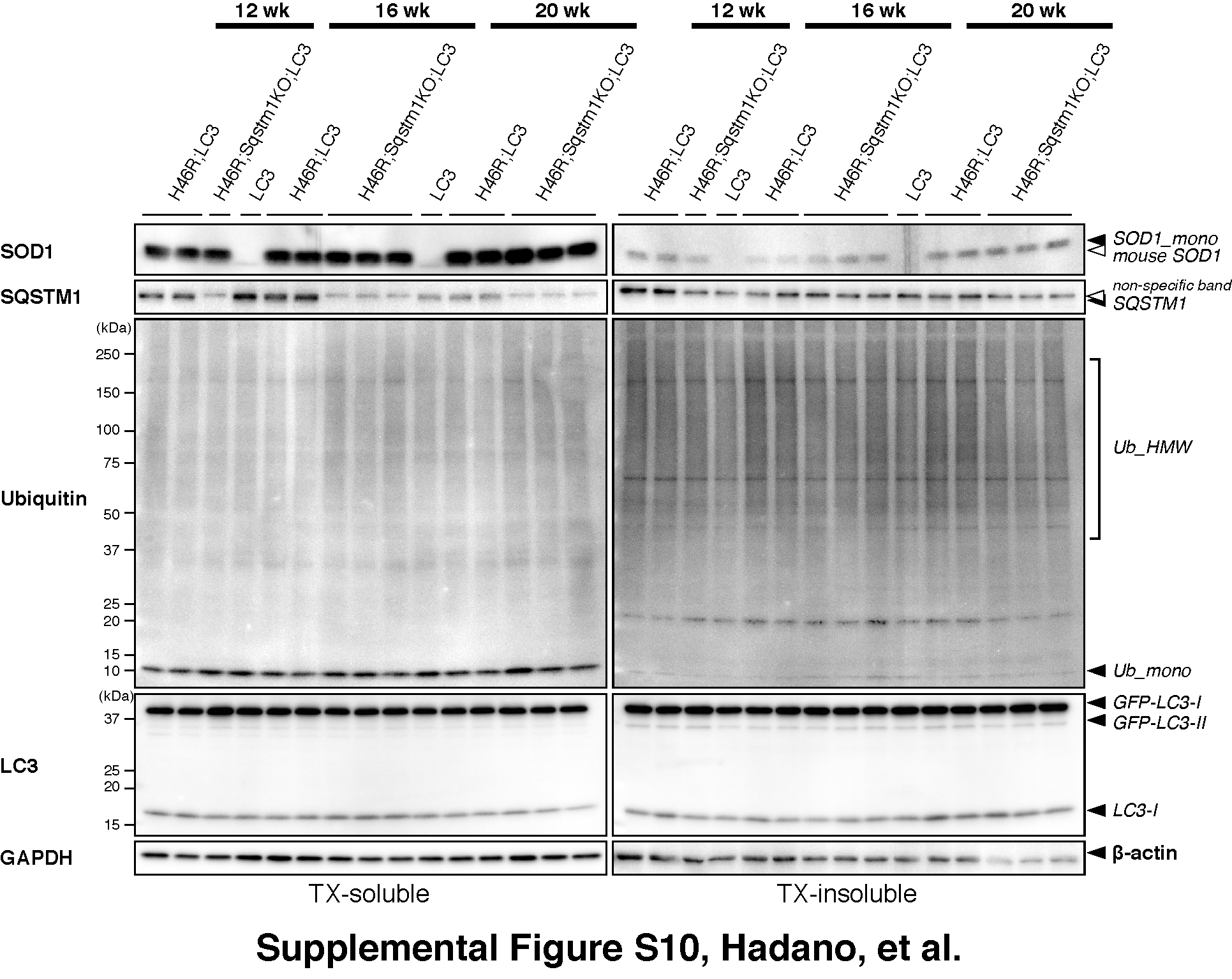

Supplement: Supplementary Data [file supp_ddw186_suppl_data.zip › FigS10.tif]
