# Supplementary material for: A recurrent p.Arg92Trp variant in steroidogenic factor-1 (NR5A1) can act as a molecular switch in human sex development
Source: Hum Mol Genet. 2016 Jul 4;25(16):3446–53. doi: 10.1093/hmg/ddw186 (PMC5179941; doi:10.1093/hmg/ddw186)
Supplement: Supplementary Data [file supp_ddw186_suppl_data.zip › Hadano_HMG_Suppl.doc]

**Supplemental Information**

**Functional links between SQSTM1 and ALS2 in the pathogenesis of ALS: Cumulative impact on the protection against mutant SOD1-mediated motor dysfunction in mice**

Shinji Hadano, Shun Mitsui, Lei Pan, Asako Otomo, Mizuki Kubo, Kai Sato,

Suzuka Ono, Wakana Onodera, Koichiro Abe, XuePing Chen, Masato Koike,

Yasuo Uchiyama, Masashi Aoki, Eiji Warabi, Masayuki Yamamoto,

Tetsuro Ishii, Toru Yanagawa, Hui-Fang Shang, and Fumihito Yoshii

**Legends for Supplementary Figures**

**Figure S1.** Gross morphology and histological analysis of long bones from wild-type (WT), *Sqstm1*^-/-^ (Sqstm1KO), *SOD1^H46R^* (H46R), and *Sqstm1*^-/-^;*SOD1^H46R^* (H46R;Sqstm1KO) mice at 20 weeks of age. (**A**) X-ray images of bone morphology of the whole body from WT (**a**), Sqstm1KO (**b**), H46R (**c**), and H46R;Sqstm1KO (**d**) mice. (**B**) H&E morphological stain of femoral long bones from WT (**e**), Sqstm1KO (**f**), H46R (**g**), and H46R;Sqstm1KO (**h**) mice. Higher magnification images of the outlined regions with white dashed lines as indicated by arrowheads are shown in the right columns (WT; **e’**, Sqstm1KO; **f’**, H46R; **g’**, and H46R;Sqstm1KO; **h’**). No structural abnormalities and differences among these mice are observed. Scale bars = 50 μm.

**Figure S2.** Nissl staining of sagittal brain sections from wild-type (WT) (**A**), *SOD1^H46R^* (H46R) (**C**), and *Sqstm1*^-/-^;*SOD1^H46R^* (H46R;Sqstm1KO) (**D**) mice at 20 weeks of age, and from *Sqstm1*^-/-^ (Sqstm1KO) mouse at 16 weeks of age (**B**). Sections of cortex (**a1**-**a4**, **b1**-**b4**, **c1**-**c4**, and **d1**-**d4**) and hippocampus (**a5**, **b5**, **c5**, and **d5**) from mice with respective genotypes are shown. No structural abnormalities and differences among mice with different genotypes are observed. Scale bars = 200 μm.

**Figure S3.** Quantitative analysis of the transcripts in the spinal cord and cerebral cortex from 12, 16, and 20 week-old mice with four distinct genotypes; wild-type (WT), *Sqstm1*^-/-^ (Sqstm1KO), *SOD1^H46R^* (H46R), and *Sqstm1*^-/-^;*SOD1^H46R^* (H46R;Sqstm1KO). The expression levels of the human *SOD1^H46R^* transgene (**A** and **B**), *Sqstm1* (SQSTM1) (**C** and **D**), *Map1lc3a* (LC3A) (**E** and **F**), *Map1lc3b* (LC3B) (**G** and **H**), *Nes* (nestin) (**I** and **J**), *Bysl* (bystin-like/bystin) (**K** and **L**), *Gfap* (glial fibrillary acidic protein) (**M** and **N**), and *Aif1* (AIF1/Iba1) (**O** and **P**) genes in the spinal cord (**A**, **C**, **E**, **G**, **I**, **K**, **M**, and **O**) and cerebral cortex (**B**, **D**, **F**, **H**, **J**, **L**, **N**, and **P**) are shown. Data are normalized by the level of *Gapdh* (glyceraldehyde 3-phosphate dehydrogenase). Values are mean ± SEM (n = 4) in an arbitrary unit relative to 12 week-old wild-type mice except for the human *SOD1* transgene expression (**A** and **B**), in which values relative to 12 week-old *SOD1^H46R^* mice are shown. Statistical significance is evaluated by two-way ANOVA with Bonferroni’s *post hoc* test (comparisons between different genotypes in the same age; **p* < 0.05, ***p* < 0.01, ****p* < 0.001, *****p* < 0.0001) or one-way ANOVA with Bonferroni’s *post hoc* test (comparisons between different ages in the same genotype; ^§^*p* < 0.05, ^§§^*p* < 0.01, ^§§§^*p* < 0.001, ^§§§§^*p* < 0.0001).

**Figure S4.** Mutant SOD1 protein-expressing mice show a progressive accumulation of vimentin and glial fibrillary acidic protein (GFAP) in the spinal cord. (**A**) Western blot analysis of vimentin and GFAP in the spinal cord from 12, 16, and 20 week-old mice with four distinct genotypes; wild-type (WT), *Sqstm1*^-/-^ (Sqstm1KO), *SOD1^H46R^* (H46R), and *Sqstm1*^-/-^;*SOD1^H46R^* (H46R;Sqstm1KO). Two fractions; 1 % Triton X-soluble fraction (TX-soluble; left panels) and 1 % Triton X-insoluble/5 % SDS-soluble fraction (TX-insoluble; right panels) are analyzed. The positions of bands representing vimentin and GFAP are shown on the right. (**B**, **C**) Quantitation of TX-insoluble vimentin (**B**) and TX-insoluble GFAP (**C**). Densitometric data for immunoreactive signals are normalized by the levels of β-actin. Values are mean ± SEM (n = 4) in an arbitrary unit relative to 12 week-old wild-type mice. Statistical significance is evaluated by two-way ANOVA with Bonferroni’s *post hoc* test (**p* < 0.05, ***p* < 0.01, ****p* < 0.001) or one-way ANOVA with Bonferroni’s *post hoc* test (comparisons between different ages in the same genotype; ^§§^*p* < 0.01, ^§§§^*p* < 0.001).

**Figure S5.** Western blot analysis of SOD1, SQSTM1, and ubiquitin (Ub) in the cerebral cortex from 12, 16, and 20 week-old mice with four distinct genotypes; wild-type (WT), *Sqstm1*^-/-^ (Sqstm1KO), *SOD1^H46R^* (H46R), and *Sqstm1*^-/-^;*SOD1^H46R^* (H46R;Sqstm1KO). Two fractions; 1% Triton X-soluble fraction (TX-soluble; left panels) and 1% Triton X-insoluble/5% SDS-soluble fraction (TX-insoluble; right panels), are analyzed. SOD1_mono and SOD1_HMW represent monomeric and high molecular-weight (aggregated) forms of SOD1, respectively. Ub_mono and Ub_HMW represent monomeric Ub and the polyubiquitinated proteins, respectively. GAPDH and β-actin are used for a loading control in TX-soluble and -insoluble fractions, respectively. The positions of size-markers are shown on the left.

**Figure S6.** Accumulation of disease-associated proteins in the spinal cord of SOD1^H46R^-expressing mice. Representative macro-view images of double immunostaining with ubiquitin (green) and SOD1 (red) in the lumbar spinal cord (L4-L5) from wild-type (WT, 1^st^ row), *SOD1^H46R^* (H46R, 2^nd^ row), *Sqstm1*^-/-^ (Sqstm1KO, 3^rd^ row), and *Sqstm1*^-/-^;*SOD1^H46R^* (H46R;Sqstm1KO, 4^th^ row) mice at 20 weeks of age. The nuclei are counterstained with DAPI (Blue). Right columns display the merged images. (**A**-**C**) Scale bars = 200 μm. (**D**) Scale bars = 100 μm.

**Figure S7.** Progressive accumulation of disease-associated proteins in the spinal cord of SOD1^H46R^-expressing mice. Representative merged images of double immunostaining with ubiquitin (green) and SQSTM1 (red) (**A**), ubiquitin (green) and SOD1 (red) (**B**), ubiquitin (green) and GFAP (red) (**C**), and MAP2 (green) and ubiquitin (red) (**D**) in the lumbar spinal cord (L4-L5) from wild-type (WT), *SOD1^H46R^* (H46R), *Sqstm1*^-/-^ (Sqstm1KO), and *Sqstm1*^-/-^;*SOD1^H46R^* (H46R;Sqstm1KO) mice at 16 and 20 weeks of ages (16wk; upper row, 20wk; lower row). The nuclei are counterstained with DAPI (Blue). Ubiquitin-positive aggregates and/or vesicular structures are observed in soma of neurons both in *SOD1^H46R^* and SQSTM1-deficient *SOD1^H46R^* mice (yellow arrowheads). It is notable that a number of ubiquitin-positive aggregates colocalizing with diffused DAPI-positive materials are observed in non-neuronal cells and/or extracellular spaces (white arrows), which may represent degenerated neuronal and/or non-neuronal cells. Scale bars = 20 μm.

**Figure S8.** Loss of SQSTM1 does not change the autophagic flux in the spinal cord of SOD1^H46R^-expressing mice. (**A**) Survival curves for *SOD1^H46R^* (H46R; filled circle; n = 49) and *SOD1^H46R^*;*GFP-MAP1LC3B* (H46R;LC3; open square; n = 35) mice. Kaplan-Meier analysis identifies no significant differences between *SOD1^H46R^* and *SOD1^H46R^*;*GFP-MAP1LC3B* mice. (**B**) Western blot analysis of LC3 in the spinal cord from 12, 16, and 20 week-old mice with three distinct genotypes; *GFP-MAP1LC3B* (LC3), *SOD1^H46R^*;*GFP-MAP1LC3B* (H46R;LC3), and *Sqstm1^-/-^*;*SOD1^H46R^*;*GFP-MAP1LC3B* (H46R;Sqstm1KO;LC3). Number of animals used varies from 1 to 3 for each genotype. Two fractions; 1 % Triton X-soluble fraction (TX-soluble; left panels) and 1 % Triton X-insoluble/5 % SDS-soluble fraction (TX-insoluble; right panels) are analyzed. GAPDH and β-actin are used for a loading control in TX-soluble and -insoluble fractions, respectively. Positions of the unmodified and lipidated forms of overexpressed GFP-LC3 are indicated as GFP-LC3-I and GFP-LC3-II, respectively. Position of the unmodified form of endogenous LC3 is also indicated as LC3-I. It is noted that the lipidated form of endogenous LC3 (LC3-II) are undetectable due to a short exposure of imaging. Size-markers are shown on the left. (**C**) The ratio of the signal intensities for LC3-II to LC3-I.

**Figure S9.** Western blot analysis of SOD1, SQSTM1, and ubiquitin (Ub) in the spinal cord from 12, 16, and 20 week-old mice with three distinct genotypes; *GFP-MAP1LC3B* (LC3), *SOD1^H46R^*;*GFP-MAP1LC3B* (H46R;LC3), and *Sqstm1^-/-^*;*SOD1^H46R^*;*GFP-MAP1LC3B* (H46R;Sqstm1KO;LC3). Number of animals used varies from 1 to 3 for each genotype. Two fractions; 1% Triton X-soluble fraction (TX-soluble; left panels) and 1% Triton X-insoluble/5% SDS-soluble fraction (TX-insoluble; right panels), are analyzed. SOD1_mono and SOD1_HMW represent monomeric and high molecular-weight (aggregated) forms of SOD1, respectively. Ub_mono and Ub_HMW represent monomeric Ub and the polyubiquitinated proteins, respectively. GAPDH and β-actin are used for a loading control in TX-soluble and -insoluble fractions, respectively. The positions of size-markers are shown on the left.

**Figure S10.** Western blot analysis of SOD1, SQSTM1, ubiquitin, and LC3 in the cerebral cortex from 12, 16, and 20 week-old mice with three distinct genotypes; *GFP-MAP1LC3B* (LC3), *SOD1^H46R^*;*GFP-MAP1LC3B* (H46R;LC3), and *Sqstm1^-/-^*;*SOD1^H46R^*;*GFP-MAP1LC3B* (H46R;Sqstm1KO;LC3). Number of animals used varies from 1 to 3 for each genotype. Two fractions; 1% Triton X-soluble fraction (TX-soluble; left panels) and 1% Triton X-insoluble/5% SDS-soluble fraction (TX-insoluble; right panels), are analyzed. SOD1_mono represents monomeric form of SOD1. Ub_mono and Ub_HMW represent monomeric Ub and the polyubiquitinated proteins, respectively. GAPDH and β-actin are used for a loading control in TX-soluble and -insoluble fractions, respectively. The positions of size-markers are shown on the left.

**Figure S11.** Western blot analysis of SOD1, SQSTM1, and ubiquitin (Ub) in the spinal cord from 12, 16, and 20 week-old mice with five distinct genotypes; wild-type (WT), *SOD1^H46R^* (H46R), *Sqstm1*^-/-^;*SOD1^H46R^* (H46R;Sqstm1KO), *Als2*^-/-^;*SOD1^H46R^* (H46R;Als2KO), and *Als2*^-/-^;*Sqstm1*^-/-^;*SOD1^H46R^* (H46R;Als2KO;Sqstm1KO). Two fractions; 1 % Triton X-soluble fraction (TX-soluble; left panels) and 1 % Triton X-insoluble/5 % SDS-soluble fraction (TX-insoluble; right panels) are analyzed. SOD1_mono and SOD1_HMW represent monomeric and high molecular-weight (aggregated) forms of SOD1, respectively. Ub_mono and Ub_HMW represent monomeric Ub and the polyubiquitinated proteins, respectively. GAPDH and β-actin are used for a loading control in TX-soluble and -insoluble fractions, respectively. The positions of size-markers are shown on the left.

**Figure S12.** Western blot analysis of vimentin and GFAP in the spinal cord from 20 week-old mice with four distinct genotypes; *SOD1^H46R^* (H46R), *Sqstm1*^-/-^;*SOD1^H46R^* (H46R;Sqstm1KO), *Als2*^-/-^;*SOD1^H46R^* (H46R;Als2KO), and *Als2*^-/-^;*Sqstm1*^-/-^;*SOD1^H46R^* (H46R;Als2KO;Sqstm1KO), and from end-stage mice with six distinct genotypes; H46R (25 week), H46R;Sqstm1KO (20 week), H46R;Als2KO (23 week), H46R;Als2KO;Sqstm1KO (20 week), *SOD1^G93A^* (G93A) (31 week), and *Sqstm1*^-/-^;*SOD1^G93A^* (G93A;Sqstm1KO) (24 week). Two fractions; 1 % Triton X-soluble (TX-sol; left panels) and 1 % Triton X-insoluble/5 % SDS-soluble (TX-insol; right panels) fractions are analyzed. GAPDH and β-actin are used for loading controls in TX-soluble and -insoluble fractions, respectively. First and second lanes in each panel represent the spinal cord samples from 23 week-old *Als2*^+/-^;*Sqstm1*^+/-^;*SOD1^H46R^* (H46R;Als2Het;Sqstm1Het) mouse, which are used for loading controls to compare the signal intensities between blots. The position of band representing GFAP is shown on the right.

**Figure S13.** Extensive accumulation of ubiquitin-positive aggregates and vesicular structures in neurons of the spinal cord from SQSTM1-, ALS2-, and ALS2/SQSTM1-deficient SOD1^H46R^ mice. (**A**) Representative images of double immunostaining with ubiquitin (green) and SOD1 (red), and (**B**) those with ubiquitin (green) and GFAP (red) in the ventral horn of the lumbar spinal cord (L4-L5) from *SOD1^H46R^* mice (H46R) at 20 weeks of age (1^st^ row) and at the end stage (25week) (2^nd^ row), *Sqstm1*^-/-^;*SOD1^H46R^* mice (H46R;Sqstm1KO) at 20 week of age (3^rd^ row) and at the end stage (21 week) (4^th^ row), *Als2*^-/-^;*SOD1^H46R^* mice (H46R;Als2KO) at the end stage (25 week) (5^th^ row), and *Als2*^-/-^;*Sqstm1*^-/-^;*SOD1^H46R^* mice (H46R;Als2;Sqstm1KO) mice at the end stage (20 week) (6^th^ row), respectively. The nuclei are counterstained with DAPI (Blue). Right columns display the merged images. Most ubiquitin-positive aggregates are observed in non-neuronal cells and/or extracellular spaces (white arrows) and are rarely seen in neurons of *SOD1^H46R^* mice, whereas SQSTM1-, ALS2-, and ALS2/SQSTM1-deficient *SOD1^H46R^* mice exhibit the preferential localization of ubiquitin-positive inclusions and/or vacuolar structures in soma of neurons (yellow arrowheads). Enlarged ubiquitin-positive dendrites and/or axons (marked as asterisk) are occasionally observed in all the mice overexpressing SOD1^H46R^ at the end stage. It is noted that ubiquitin-positive extracellular large vacuolar structures are also observed in ALS2-deficient *SOD1^H46R^* mice (open arrowheads). Scale bars = 20 μm.

**Legend for Supplementary Table**

**Table S1.** Summary of the lifespan data analyzed in the present study.
